# Limited functional conservation of a global regulator among related bacterial genera: Lrp in *Escherichia*, *Proteus *and *Vibrio*

**DOI:** 10.1186/1471-2180-8-60

**Published:** 2008-04-11

**Authors:** Robert E Lintner, Pankaj K Mishra, Poonam Srivastava, Betsy M Martinez-Vaz, Arkady B Khodursky, Robert M Blumenthal

**Affiliations:** 1 Department of Medical Microbiology and Immunology, University of Toledo Health Sciences Center, Toledo, OH 43614-2598, USA; 2 Department of Biochemistry, Molecular Biology, and Biophysics, University of Minnesota, St. Paul, MN 55108-1022, USA; 3 Biotechnology Institute, University of Minnesota, St. Paul, MN 55108-1022, USA; 4 Program in Bioinformatics and Proteomics/Genomics, University of Toledo Health Sciences Center, Toledo, OH 43614-2598, USA; 5 Current Address: Whitehead Institute for Biomedical Research, 9 Cambridge Center, Cambridge, MA 02142, USA; 6 Current Address: Department of Pediatrics, Wayne State University, Detroit, MI 48201, USA; 7 Current Address: Hamline University, St. Paul, MN 55104, USA

## Abstract

**Background:**

Bacterial genome sequences are being determined rapidly, but few species are physiologically well characterized. Predicting regulation from genome sequences usually involves extrapolation from better-studied bacteria, using the hypothesis that a conserved regulator, conserved target gene, and predicted regulator-binding site in the target promoter imply conserved regulation between the two species. However many compared organisms are ecologically and physiologically diverse, and the limits of extrapolation have not been well tested. In *E. coli K-*12 the leucine-responsive regulatory protein (Lrp) affects expression of ~400 genes. *Proteus mirabilis *and *Vibrio cholerae *have highly-conserved *lrp *orthologs (98% and 92% identity to *E. coli lrp*). The functional equivalence of Lrp from these related species was assessed.

**Results:**

Heterologous Lrp regulated *gltB*, *livK *and *lrp *transcriptional fusions in an *E. coli *background in the same general way as the native Lrp, though with significant differences in extent. Microarray analysis of these strains revealed that the heterologous Lrp proteins significantly influence only about half of the genes affected by native Lrp. In *P. mirabilis*, heterologous Lrp restored swarming, though with some pattern differences. *P. mirabilis *produced substantially more Lrp than *E. coli *or *V. cholerae *under some conditions. Lrp regulation of target gene orthologs differed among the three native hosts. Strikingly, while Lrp negatively regulates its own gene in *E. coli*, and was shown to do so even more strongly in *P. mirabilis*, Lrp appears to activate its own gene in *V. cholerae*.

**Conclusion:**

The overall similarity of regulatory effects of the Lrp orthologs supports the use of extrapolation between related strains for general purposes. However this study also revealed intrinsic differences even between orthologous regulators sharing >90% overall identity, and 100% identity for the DNA-binding helix-turn-helix motif, as well as differences in the amounts of those regulators. These results suggest that predicting regulation of specific target genes based on genome sequence comparisons alone should be done on a conservative basis.

## Background

Microbial genome sequences are being determined with increasing frequency and speed. Nearly 500 bacterial genomes have been fully sequenced, and nearly 2000 more such projects are underway [1], with several current and planned large-scale metagenomic projects adding to the gathering avalanche of data [2-10]. A major motivation for this sequencing avalanche is the possibility of learning about a bacterium's physiology or pathogenesis, without resorting to either labor-intensive classical analyses or the still-expensive tools of systems biology. Increasingly effective methods are available to generate a "parts list" of genes and pathways from genome sequences of poorly-characterized bacteria [11-14]. However understanding the physiology of an organism, in terms of gene regulatory mechanisms and network connections, is currently much more difficult to achieve from sequence analysis alone.

Considerable research is focused on inferring gene regulatory networks from microarray analyses, following genetic or environmental disturbances [15-19]. However some microbes, for which the genome sequence is available from metagenomic studies, cannot even be grown in the laboratory. Particularly for poorly understood bacteria, it is unclear which experimental disturbances would be most physiologically relevant and would meaningfully probe the regulatory architecture [20].

Predicting gene regulatory networks from genome sequences alone is difficult, but can yield useful hypotheses about the conservation and evolution of regulatory networks [21-24]. Such prediction is typically accomplished by extrapolating from a well-characterized reference organism such as *E. coli*, if three criteria can be satisfied. The first two criteria are whether valid orthologs for a target (regulated) gene and its regulator (in the reference organism) are present in both organisms. Though it is unclear exactly *how *similar orthologous sequences must be for functional and regulatory predictions to have a sufficiently high probability of being accurate [25-29], these determinations are relatively straightforward from a computational perspective.

The third criterion, identifying a putative binding site for the regulator upstream of the orthologous target gene, is more complex [30-37]. Among other problems, many regulators have degenerate binding motifs and commonly-used approaches have limited sensitivity and specificity [38,39]. In addition, the relative strength of a binding site can be as important to the resultant regulatory pattern as the site's existence [40], but binding strength is difficult to predict from sequence alone [41,42], especially where the structure of the regulator is unknown [43,44]. These difficulties have led some bioinformatic analyses to focus on the regulator-target gene connection alone, without attempting to predict the sign or strength of the interaction, and such approaches can provide useful information, if limited from the perspective of predicting or modeling cell physiology.

An even more basic issue is how similar two bacterial species have to be for the underlying hypothesis, which for brevity we refer to as the "regulatory extrapolation hypothesis," to be usefully applied. Even if orthologous genes and binding sites could be identified unambiguously, to what extent *do *a matching regulator, target gene and binding site correctly predict regulation? There are several potential concerns that, while not ruling out extrapolation, could place limits on its applicability. First, regarding the orthologous regulators, extrapolation implicitly assumes that they are similar in both DNA binding and in response to coregulators (if any). Second, it is implicitly assumed that the *amounts *of both regulator and coregulator vary in similar ways in the two organisms. Third, regarding the promoter regions for the orthologous target genes, regulatory patterns are flexible [45-47] and can be profoundly changed by limited mutation. For example, a single nucleotide change in the *soxS *promoter results in repression by SoxR, which normally activates, regardless of the redox signal [48]; and just 1–2 amino acid replacements in a regulatory protein can alter the range of coactivators or change the effect of an inducer to that of a corepressor [49,50]. Fourth, some regulatory extrapolations involve species that are, ecologically at least, quite different from one another, and (not surprisingly) the environment to which an organism is adapted affects its regulatory architecture [51]. Developing more robust computational approaches requires a fuller experimentally-based understanding of the extent to which regulatory architecture is conserved among related bacteria adapted to different environments.

The aim of this study is to assess conservation of regulatory architecture by studying a model regulator, and the network of target genes it controls (its "regulon"), in three related bacteria with fully-sequenced genomes. *E. coli K*-12 is the well-studied reference organism [52,53]. *Proteus mirabilis *is, like *E. coli*, among the Enterobacteriaceae, but is a relatively distant member of that family [54]. *Vibrio cholerae *is a member of a different family within the γ-Proteobacteria – the Vibrionaceae [55]. These organisms share some basic properties. All three grow on mucosal epithelia, and all three are capable of differentiating into elongated, hyperflagellated swarmer cells that spread across solid surfaces [56-60]. *E. coli*, however, is adapted to growth in the mammalian or avian intestine, while *P. mirabilis *is a urinary tract pathogen, and *V. cholerae *is primarily a marine microbe that is an opportunistic pathogen of the human ileum [61].

**Figure 1:**
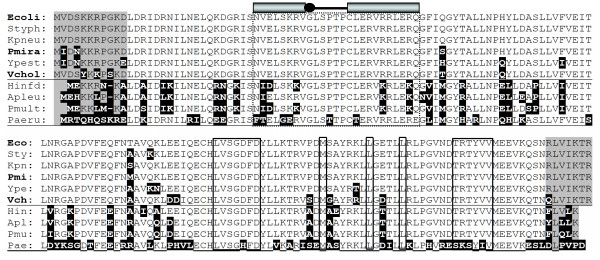
**Sequences of selected Lrp proteins.** Lrp proteins from various bacterial species were aligned; species used in this study are in bold (*Escherichia coli*, *Proteus mirabilis*, and *Vibrio cholerae*). A more complete list of Lrp orthologs and paralogs can be found online [140]. The gray-shaded regions indicate N- and C-terminal sequences that are conserved among enterobacterial Lrp orthologs, and the black-shaded regions indicate substitutions relative to *E. coli*. The boxed regions indicate the DNA-binding helix-turn-helix motif (top portion, under cartoon representation), and the leucine-binding sites (lower portion of sequence); all boxed regions are completely conserved among the species used in this study. For references, see main text. Other Lrp orthologs shown came from (in order shown): *Salmonella enterica *serovar Typhimurium, *Klebsiella pneumoniae*, *Yersinia pestis*, *Haemophilus influenzae *Rd, *Actinobacillus pleuropneumoniae*, *Pasteurella multocida*, and *Pseudomonas aeruginosa*.

A bacterium's transcriptional regulatory architecture is particularly dependent on its "global regulators". The number of genes controlled by a given transcription factor follows a power law distribution, and in *E. coli *about half of all genes are responsive to one or more of seven key global regulators [62]. Any meaningful understanding of an organism's gene regulation requires an understanding of the roles played by its global regulators. For this reason, we chose as our model one of the seven key regulators: Lrp, the leucine-responsive regulatory protein [63-65]. Lrp is highly conserved among the Enterobacteriaceae and Vibrionaceae (see Fig. 1, top six sequences in each panel, and Fig. S1). The Lrp regulon has been extensively mapped in *E. coli *by microarray analyses, two-dimensional gel electrophoresis, and *lacZ *fusion libraries [66-70]. The microarray analyses revealed that Lrp influences the expression of nearly 400 genes, at least 70 of which are directly controlled (ABK, unpubl. data). The RegulonDB database [71,72] currently recognizes 57 genes as being directly controlled by Lrp, based on literature surveys.

The Lrp regulon is a good model for comparison among species for at least three reasons. First, the regulon is large and includes genes having a range of functions (including biosynthesis, catabolism, transport and virulence). Second, Lrp can generate diverse regulatory patterns that include both activation and repression, and differing sensitivities to the coregulators L-leucine and L-alanine [73,74]. Third, while Lrp is abundant (2,500 molecules/cell) compared to many other transcription factors, it is present at much lower concentrations than the major nucleoid-structuring proteins Fis (60,000 molecules/cell), HU (30,000–55,000) and H-NS (20,000; all during exponential growth) [75]. Despite the ability of Lrp to bind DNA semispecifically [76], expression of the great majority of genes is unaffected by deletion of *lrp *(see Fig. 1A in [69] and Fig. 3 in [68]), so its generalized effects as a histonelike protein are limited.

To compare the architecture of the Lrp regulons of *E. coli*, *P. mirabilis*, and *V. cholerae*, we began by addressing four general questions. First, are the effects of *lrp *disruption on complex phenotypes such as growth and swarming comparable in the three species? Second, are the *lrp *genes functionally interchangeable in complementation assays? Third, do the *lrp *genes themselves have the same expression pattern in the different species? Fourth, are the orthologs of what, in *E. coli K-*12, are Lrp-controlled genes regulated by Lrp in the same manner in the two other species? The goal of this study was not to determine the molecular bases for observed differences, but rather to assess the frequency of such differences.

## Results

### Lrp is highly conserved among Enterobacteriaceae and Vibrionaceae

It would be extremely useful if transcriptional regulatory architecture of bacteria could be predicted from the sequence of their DNA. Attempts to do so generally involve extrapolation from well-studied species such as *E. coli*, in cases where the regulators and target genes are orthologous and a binding site is conserved in the target promoter. However, such extrapolation relies on several implicit assumptions (see Introduction) that have not been well tested experimentally. We used the Lrp regulon as a model to carry out tests of these assumptions.

The pronounced conservation among Lrp orthologs in enteric bacteria was first noted over a decade ago [77], and the large number of subsequently-determined genome sequences has not altered that pattern. The Lrp ortholog in *P. mirabilis *differs from that in *E. coli *by only 4/164 amino acids (98% identity), while Lrp from *V. cholerae *shows 92% identity to *E. coli *Lrp. Importantly, none of the changes observed in *P. mirabilis *and *V. cholerae *occur in the helix-turn-helix motif responsible for DNA sequence recognition (Fig. 1, cartoon representation and boxed region), defined via mutation of the *E. coli *lrp gene and x-ray crystallography of an archaeal ortholog [78,79] and recently of the *E. coli *protein itself [80]. Similarly, the Lrp orthologs of these bacteria are completely conserved for amino acids implicated in coregulator recognition (boxed amino acids in lower panel of Fig. 1).

Lrp orthologs from another γ-proteobacterial family, the Pasteurellaceae (including *Haemophilus influenzae *and *Pasteurella multocida*), are much more divergent from *E. coli *(Fig. 1), with the differences specifically including the helix-turn-helix motif. It is interesting that the Pasteurellaceae appear to form an outgroup with respect to Lrp, as the Lrp differences are far more pronounced than their overall relationship to neighboring bacterial genera would suggest (see Fig. 2 in [81], and Fig. S1 in Additional file 1). This, and the fact that in *H. influenzae *Lrp controls only a small number of genes [82], led us to exclude the Pasteurellaceae from this study.

**Figure 2:**
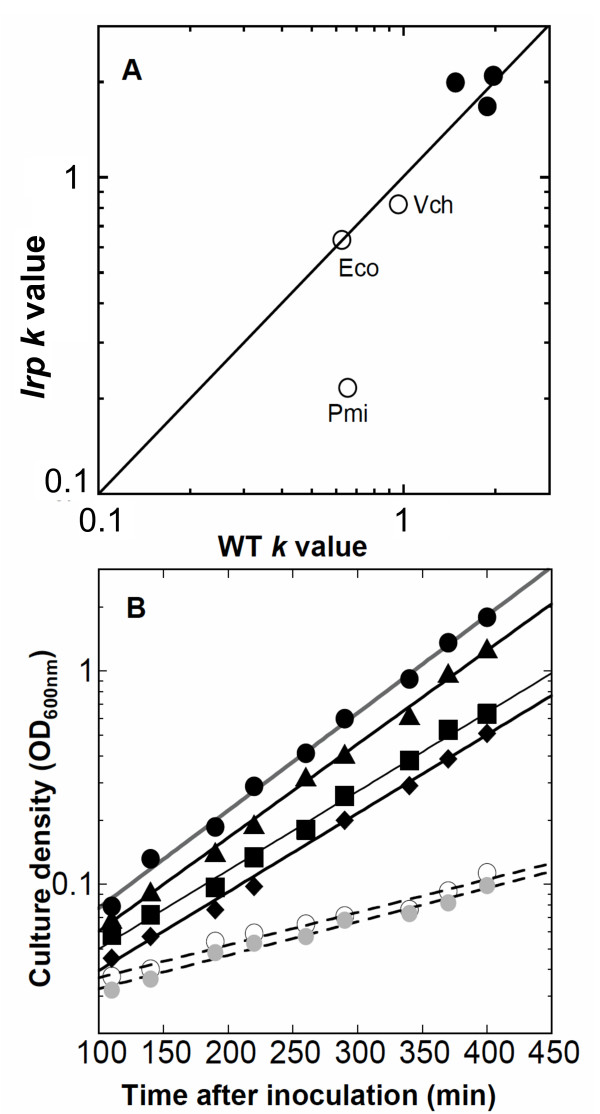
**Effects of *lrp *null mutation on growth rates.** Growth rates were determined from a fit to the exponential portion of the growth curve, extending in all but one case (*P. mirabilis*, glucose minimal medium) through at least four mass doublings. Open symbols refer to growth in MOPS glucose plus required supplements (nicotinate, panthothenate and thiamine; see Methods), while closed symbols represent growth in MOPS glucose defined-rich medium. **A**. *lrp vs. lrp*^+^growth rates. The values shown are the specific growth rate constants, *k*, calculated as ln2/(doubling time, in h). For comparison, *k *values of 0.5, 1, and 2 correspond respectively to doubling times of 83, 42, and 21 min. The rich medium results are clustered and therefore not labeled; for the minimal medium, the abbreviations used are Eco (*E. coli*), Pmi (*P. mirabilis*), and Vch (*Vibrio cholerae*). The diagonal line shows where points should fall if *lrp *mutation has no effect on the growth rate in these media. **B**. Complementation of the low *P. mirabilis *growth rate in the glucose minimal medium described in (A). The dashed lines indicate growth data for the *P. mirabilis lrp *mutant (open circles; 193 min doubling time) and the mutant bearing the vector control (gray circles; 191 min). Remaining lines show the WT *P. mirabilis *(closed circles; 66 min); and the *lrp *mutant bearing plasmids with the *lrp*^+ ^genes from *P. mirabilis *(triangles; 69 min), *V. cholerae *(squares; 81 min), or *E. coli *(diamonds; 81 min).

### Differences in growth phenotypes of *lrp *null strains

The first test of functional conservation is that if Lrp is having similar broad effects on gene expression in *E. coli*, *P. mirabilis*, and *V. cholerae*, then one would expect to see similar effects of a *lrp *null mutation on their growth. Fig. 2A shows the results of growth experiments for wild-type (WT) and *lrp *strains of these three species grown in MOPS glucose minimally supplemented medium ("MOPS glucose") or MOPS glucose defined rich medium ("MOPS rich") [both media including nicotinate as required by the *P. mirabilis *strains, and pantothenate and thiamine as required by the *P. mirabilis *Δ*lrp *strain (see Methods)]. The plot shows the WT specific growth rate on the x-axis, and the rate for the *lrp *strain on the y-axis; thus where *lrp *mutation has no effect on growth rate the points fall on the diagonal line. In MOPS rich medium (closed symbols), *lrp *mutation had little effect on growth of any of the three species. However in MOPS glucose medium, *P. mirabilis *stands out as having a substantial growth rate decrease when *lrp *is mutated (193 min *vs*. 66 min doubling time for the WT strain). This might represent a *lrp*-dependent partial auxotrophy, in addition to the *lrp-*dependent requirements for pantothenate and thiamine that were satisfied by the medium. However that may be, it is clear that the *lrp *mutation has differential effects in these three species.

We cloned the *lrp *genes from *P. mirabilis *and *V. cholerae *downstream of P*lacUV5 *in the low-copy vector pCC1 (Epicentre); a consensus *E. coli *Shine-Dalgarno sequence [83] was introduced as well. We also cloned the *lrp *gene from *E. coli *O157:H7 (which is identical to that of *E. coli K*-12 at the amino acid level). Thus the three *lrp *alleles had identical expression sequences. The effect of the *lrp *allele on *P. mirabilis *growth in minimal medium was fully complemented by supplying the cloned *P. mirabilis lrp *gene on a plasmid, and was mostly but incompletely complemented by the *lrp *genes from *E. coli *and *V. cholerae *(Fig. 2B).

### Effects of orthologous *lrp *alleles on swarming behavior in Proteus

**Figure 3:**
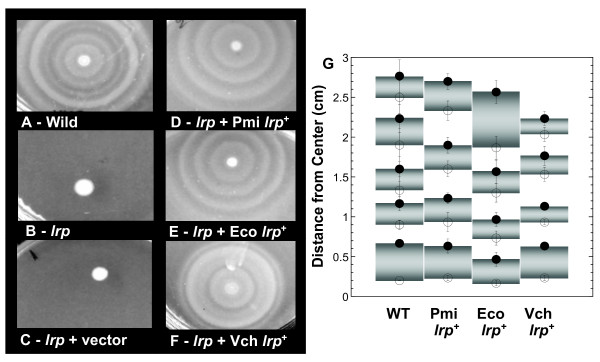
**Effect of heterologous Lrp proteins on the swarming phenotype of *P. mirabilis*.***Proteus mirabilis *wild-type (A) or *lrp *null strains (B) were grown in LB medium. Overnight cultures were spotted (2 μl) onto triplicate 1.5% agar LB plates. After 12 h at 37°C, plates were photographed under normal illumination. At the same time, transformants of the *lrp *mutant strain were assayed in parallel using the same methods. These strains contained pCC1 vector (C), or plasmids carrying *lrp*^+ ^alleles from *P. mirabilis *(D), *E. coli *(E), or *V. cholerae *(F). Panel G shows the results of measurements (average ± standard error of the triplicates) from the center of each colony to the inner (open) and outer (filled) edges of the growth rings.

We next tested the ability of Lrp orthologs to complement a complex phenotype other than growth. *P. mirabilis *undergoes differentiation to form hyperflagellated swarmer cells >20-fold longer than nonswarmer cells, and yields concentric rings of growth on agar [56,59]; Fig. 3A). Like growth rate, swarming is sensitive to a variety of factors and thus also provides a sensitive indication of the cell's physiological status [59]. It has been shown by others [84] that a *lrp *mutation abolishes swarming in *P. mirabilis *(Fig. 3B). We show here that the *lrp *orthologs from both *E. coli *and *V. cholerae *complement a *P. mirabilis lrp *mutant, and restore the complex swarming behavior (Fig. 3, panels E and F). The *P. mirabilis lrp *gene does not regenerate the exact WT swarming pattern when supplied *in trans *(Fig. 3A, D); this difference may reflect replacement of the native *lrp *expression sequences with P*lacUV5 *on the plasmid. However, despite the fact that the three plasmid-borne *lrp *alleles had identical expression sequences, they gave consistent differences in the *P. mirabilis *swarming patterns as reflected in growth ring measurements from triplicate experiments (Fig. 3G). In this experiment, only the regulator (Lrp) was varied; the *P. mirabilis *target genes and promoters/binding sites are identical between strains. Thus the significant differences in swarming (*e.g*., comparing the outer growth rings of strains complemented by *lrp *from *Vibrio *and *Proteus *in Fig. 3G) indicate functional differences in the Lrp proteins, some of which may be amplified by indirect effects.

### Regulation of *E. coli *target genes by Lrp proteins from *P. mirabilis *and *V. cholerae*

**Figure 4:**
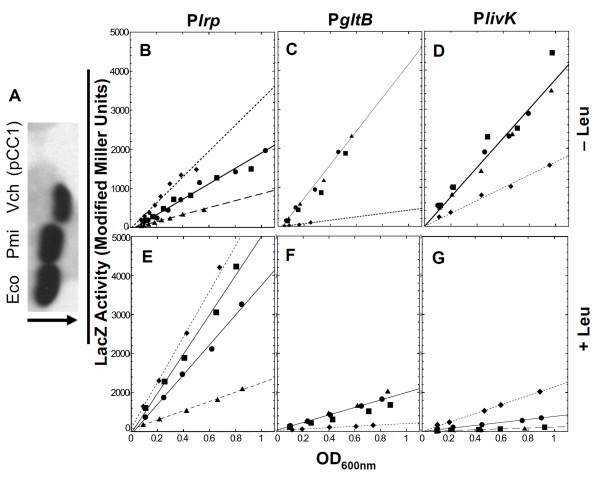
**Regulation of selected target genes by heterologous Lrp proteins.***E. coli *strains, all carrying *lrp-Tn*10 and Δ*lac*, were transformed with plasmids carrying various *lrp *alleles (or vector control). Transformants were grown in unsupplemented MOPS glucose medium. **A**. Western blot analysis of Lrp accumulation (Eco, *E. coli *Lrp; Pmi, *P. mirabilis *Lrp; Vch, *V. cholerae *Lrp; pCC1, vector control) using polyclonal antiserum raised against *E. coli *Lrp. The arrow indicates the direction of electrophoresis. **B-D**. P*lrp-lacZ *(B), P*gltB-lacZ *(C) and P*livK-lacZ *(D) activity were measured via ONPG hydrolysis, and plotted *vs*. culture density to ensure that the cultures were in balanced growth. The Lrp orthologs used are from *P. mirabilis *(triangles) and *V. cholerae *(squares), as well as the *E. coli *positive control (circles) and the vector control (diamonds). **E-G**. Isoleucine, Leucine and Valine was added to the medium ("+Leu") for experiments depicted in the lower panels: *Plrp-lacZ *(E), *PgltB-lacZ *(F) and *PlivK-lacZ *(G). The correlation coefficients for the least-squares fits to the data were all at least 0.97.

If closely-related Lrp orthologs are fully functionally conserved, as would be necessary for regulatory extrapolation, then the orthologous WT *lrp *alleles should cross complement to generate the same pattern of target gene regulation. We therefore tested the ability of heterologous Lrp proteins to properly regulate three Lrp-responsive genes in a *lrp *null mutant of *E. coli*. The *lrp*-bearing plasmids described above, which produce Lrp independently of the normal growth-dependent control associated with the native *lrp *gene [85,86], were used to transform *E. coli *strains containing reporter fusions to known Lrp target genes. Western blot analysis of a constant amount of total protein, probed with a polyclonal anti-Lrp antiserum [87], revealed comparable accumulation of the various Lrp orthologs (Fig. 4A). The strain carrying the *V. cholerae lrp *gene appeared to accumulate ~75% as much Lrp protein as the *E. coli *control (Fig. 4A and data not shown), though this is a minimal estimate because the antiserum was generated against *E. coli *Lrp (92% identical to *V. cholerae *Lrp at the amino acid level). It is interesting that the Vibrio Lrp migrated slightly faster than the other Lrp proteins. Its calculated mass (18.79 kDa) is slightly smaller than that of the other two proteins (18.89 and 18.92), and its calculated pI is less basic (7.7 *vs*. 8.9 for both of the others); other factors can also affect migration of individual polypeptides in SDS acrylamide gels [88].

To begin testing the functional equivalency of the different Lrp orthologs, we co-transformed strain BE10.2 (Δ*lacZ*, *lrp-Tn*10) with a vector containing an *E. coli *P*lrp-lacZ *fusion and the various *lrp*-bearing plasmids (or vector control). Lrp directly represses its own promoter in *E. coli*, and this occurs whether or not leucine is present [89]. Strains were grown in MOPS glucose, and the specific activity of β-galactosidase was determined and plotted against culture density to more quantitatively assess its level and to assure that the cultures were in balanced growth. Compared to the vector control, *V. cholerae *Lrp repressed P*lrp-lacZ *to the same extent as *E. coli *Lrp (~2 fold), while *P. mirabilis *Lrp (98% identical to *E. coli *Lrp) repressed about twice that much (Fig 4B).

Transcriptional activation is generally a more demanding process than repression, in the sense that the activator has not only to bind the DNA correctly but also (in most cases) to make productive contacts with RNA polymerase [90,91]. Strain BE3780 contains a chromosomal operon fusion to the gene for glutamate synthase (*gltB-lacZ*) in the *E. coli *BE10 background (Δ*lacZ*, *lrp-Tn*10) [74]. Lrp directly and strongly activates *gltBD *transcription in *E. coli *[74,92], in a process that also requires the global regulator IHF [93] and is antagonized by Crp and ArgR [94]. We found that, relative to the vector control, activation of *gltB *transcription by *P. mirabilis *or *V. cholerae *Lrp was essentially indistinguishable from that of the *E. coli lrp *positive control (Fig. 4C).

### Leucine responsiveness of heterologous Lrp proteins

Conserved function among regulators depends not only on DNA-binding (and RNA poymerase-contacting) properties, but in some cases also on responses to small molecule coregulators. This provides the basis for a third test of assumptions in regulatory extrapolation. Lrp transduces metabolic signals in the form of amino acid pool levels, in particular the amino acids L-leucine and L-alanine [64]. The *livKHGMF *operon is one of two high-affinity branched chain amino acid transport systems in *E. coli *[95]. The *livK *gene is repressed by Lrp when exogenous leucine is present [70,96], and activated when leucine is not in the medium [66]. Thus *livK *is a particularly sensitive indicator of the responses of Lrp orthologs to leucine. The amino acid residues previously demonstrated to be involved in leucine binding in *E. coli *Lrp [64,97] are completely conserved in the *P. mirabilis *and *V. cholerae *orthologs (boxes in lower half of Fig. 1).

We prepared a P*livK-lacZ *operon fusion (pRLIV2), and co-transformed it into *E. coli *BE10.2 (Δ*lacZ*, *lrp-Tn*10) together with plasmids bearing the heterologous *lrp *alleles under the control of P*lacUV5 *(Table 1). These strains were grown in MOPS glucose media containing isoleucine (I, Ile) and valine (V, Val), with or without leucine (L, Leu). Leu was not used alone as it can lead to starvation for Ile, via feedback inhibition of L-threonine deaminase [98]. β-galactosidase activity was determined as described above.

**Table 1:** Bacterial strains and plasmids used.

**Strains**	**Description**	**Source**
*Escherichia coli *W3110	F^- ^prototroph	F.C. Neidhardt
*E. coli *BE10.2	*W3110 Δlac-169 lrp35::Tn10*	R.G. Matthews
*E. coli *BE3780	*lrp::Tn10, Δlac-169, gltB(psiQ35)::lacZ*	R.G. Matthews
*E. coli *EPI300	F^- ^*mcrA *Δ (*mrr-hsdRMS-mcrBC*) Φ80d *lacZΔM15 *Δ*lacX74 recA1 endA1 araD139 *Δ (*ara, leu*)*7697 galU galK *λ^- ^*rpsL nupG trfA dhfr*	Epicentre
PS2209	W3110 Δ*lac-169*	F. C. Neidhardt
MG1655	F-lambda-*ilvG*-*rfb*-50 *rph*-1	ATCC 700926
MG1655 Δ*lrp*	F-lambda-*ilvG*-*rfb*-50 *rph*-1 Δ*lrp*::kan	This work
*Proteus mirabilis *HI4320	WT	H.L. Mobley
*P. mirabilis *U6450	WT	G.M. Fraser
*P. mirabilis *U6450*Δlrp*	*lrp::Tn5*	G.M. Fraser
*Vibrio cholerae El tor *A1552	WT	G.K. Schoolnik
*V. cholerae El tor *A1552*Δlrp*	*lrp-cat *derivative of strain A1552	G.K. Schoolnik

**Plasmids**		

pCC1	Low copy blunt cloning vector	Epicentre
pECLRP	pCC1 backbone with *lrp *gene from *E. coli O157:H7 *inserted at BamHI site	This work
pPMLRP	pCC1 backbone with *lrp *gene from *P. mirabilis HI4320 *inserted at BamHI site	This work
pVCLRP	pCC1 backbone with *lrp *gene from *V. cholerae El tor A1552 *inserted at BamHI	This work
pVEC	pCC1 backbone with Kan cassette cloned into BamHI site; used as vector control.	This work
pRLIV2	pACYC backbone with *livK-lacZ *transcriptional fusion. CAT gene was inactivated by digesting with NcoI, in-fill with Klenow fragment and religated to create a frame shift	This work
pECKAN	pECLRP with CAT gene deleted and KAN cassette inserted at BsmI	This work
pPMKAN	pPMLRP with CAT gene deleted and KAN cassette inserted at BsmI	This work
pVCKAN	pVCLRP with CAT gene deleted and KAN cassette inserted at BsmI	This work
pVEC2	pVEC with CAT gene deleted and KAN cassette inserted at BsmI	This work
pPM2005	pBH403 backbone with *E. coli O157 gltB *promoter cloned at BamHI to SalI	[93]
pPM2007	pBH403 backbone with *Proteus mirabilis gltB *promoter cloned at BamHI to SalI	This work
pPM2008	pBH403 backbone with *Vibrio cholerae *Vc2376*gltB-2 *promoter cloned at BamHI to SalI	This work

pPM2009	pBH403 backbone with *Vibrio cholerae *Vc2373*gltB-1 *promoter cloned at BamHI to SalI	This work
pPM3001	pBH403 backbone with *E. coli W3110 lrp *promoter region cloned at BamHI to SalI	This work
pPM3003	pBH403 backbone with *Proteus mirabilis lrp *promoter cloned at BamHI to SalI	This work
pPM3006	pBH403 with *Vibrio cholerae lrp *promoter cloned at BamHI to SalI	This work
pPM3001Chl	pPM3001 CAT gene was inactivated by digesting with with NcoI, in-fill with Klenow and religated to create a frame shift	This work

In general the Lrp proteins from *P. mirabilis *and *V. cholerae *yielded *livK *regulatory patterns similar to that of the *E. coli *control, showing activation in the absence of leucine and repression in its presence (Fig 4D and 4G). However both *P. mirabilis *and *V. cholerae *Lrp gave threefold greater repression than *E. coli *Lrp (Fig 4G). We also looked at the effects of adding ILV on the regulation of *E. coli *P*lrp *and P*gltB*. Addition of ILV interfered to a moderate extent with the repression of P*lrp *by all three Lrp orthologs (Fig 4E), but the differences observed in the MOPS glucose cultures were maintained, with substantially greater repression by *P. mirabilis *Lrp. Also as expected from previous studies [74,92], activation of P*gltB *by *E. coli *Lrp was moderately reduced in the presence of ILV; the two heterologous Lrp orthologs gave patterns essentially indistinguishable from that of *E. coli *Lrp (Fig 4F).

### Microarray analysis of gene regulation by heterologous Lrp proteins – effects on regulon membership

The functional conservation of Lrp orthologs was more broadly assessed by using microarrays to analyze gene regulation, when *lrp *alleles from *E. coli*, *P. mirabilis*, or *V. cholerae *(all fused to the same expression sequences) were used to complement the *lrp *null mutation in *E. coli *K-12. In these experiments, the only variable is the Lrp itself – the target genes and promoter binding sites are identical. The data for all significantly-affected genes are available [see Additional file 2].

**Figure 5:**
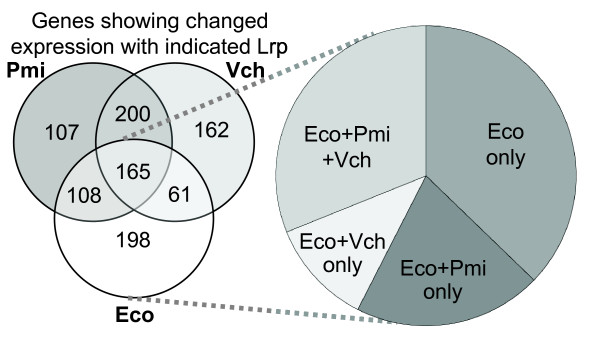
**Genome-wide comparison of transcriptional effects of three Lrp orthologs.** The Venn diagram shows subsets of *E. coli *genes that were differentially regulated in response to Lrp orthologs from the indicated species (but not to the vector control). Gene expression was assessed by two-color microarray analysis as described in Methods. The pie chart represents the relative distribution of genes significantly responsive to the *E. coli *Lrp that are also significantly responsive to the other Lrp orthologs. Details of the statistical analysis of these data are in Methods, and the gene-specific results are available [see Additional file 2].

Only 16% of the genes differentially regulated by one or more of the three Lrp orthologs were regulated in common by all three of them (Fig. 5), but this group included recognized Lrp-controlled genes such as *gltBD*, *ilvG1*, *lysU*, and *osmC *[67,74,99-101] (these results are available in spreadsheet form [see Additional file 2]). Roughly half of the genes regulated by *E. coli *Lrp under these conditions, directly or indirectly, were regulated by either of the other two orthologs (51% by *P. mirabilis *Lrp, and 42% by *V. cholerae *Lrp). Similar proportions were seen when activated and repressed genes were considered separately (not shown). [Note: "repression" and "activation" as used here include both direct and indirect effects.]

A potential concern with these experiments is that some apparent differences between the gene sets responsive to the three Lrp orthologs might be artifactual. For example, if the typical significantly-affected gene varies twofold, but this change is only counted as being significant in half of the genes that truly change twofold, then 1/4 of genes would appear to be unaffected by a Lrp ortholog, even if there were no real functional differences between the Lrp proteins. We addressed this concern in two ways.

First, the NULL hypothesis for observed overlaps under our experimental design is that two different Lrp proteins should produce identical (100% overlapping) sets of differentially-expressed genes. We are not aware of any distribution test statistic that can be used to evaluate this hypothesis (if the NULL hypothesis had been that two sets are random, then we could have used either hypergeometric distribution or Fisher's exact test). Instead we performed a bootstrap procedure to enumerate all possible outcomes of overlaps, given the data. If our actual NULL hypothesis were true, two complementation comparisons (*e.g*. *E. coli *Lrp vs. *V. cholerae *Lrp) would be indistinguishable. To test this, we generated a NULL distribution of overlaps by randomly exchanging columns of ratio values between two sets.

**Figure 6:**
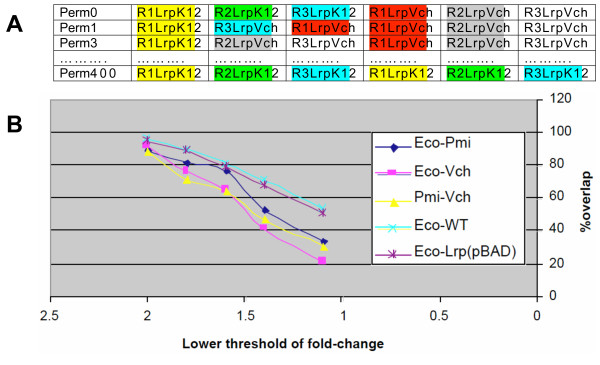
**Statistical tests for significance of microarray results.** For details, see text. **A**. Generating a NULL distribution of overlaps by randomly swapping columns of ratio values between two sets for the data that were used to generate Figs. 5 and 7. For each gene in a comparison between (for example) the *E. coli *and *V. cholerae *Lrps, there are three ratios (R1–3) for LrpK12 and three for LrpVch. The first three ratios are from the triplicate experiments in which the *lrp *null *E. coli *strain was complemented with a plasmid carrying the *E. coli lrp*^+ ^gene (LrpK12/*lrp*), and the second three ratios are from the complementations with *V. cholerae lrp *(LrpVch/*lrp*). **B**. Comparison of the fractions of genes differentially regulated (directly or indirectly) by both Lrps in pairwise comparisons. The analysis was carried out using different ratio cut-offs, among the sets presented in Fig. 7. Fractions were calculated as the number of common genes in two sets (having an expression ratio above a given limit), divided by the total number of unique genes in both sets (above the same limit).

Each set contains three ratio values (R1–3), as a result of the experiments having been carried out in triplicate. For each set we determined differentially-expressed genes at α = 0.05 using a *t*-test, and then determined the size of the intersection. Next, we "permuted" the sets as shown in Fig. 6A. There are 20 combinations in which three ratios can be selected out of six. Thus $C_{3}^{6}xC_{3}^{6}$ pairs of sets were *t*-tested, "differentially-expressed" genes were identified, and the number of such genes in the intersection was determined. The number of times the size of the overlap is greater than that observed in the real comparison, divided by 400 (the number of "permutations") indicates the significance of the observed overlap – effectively a simulated p-value. Table 2 shows the results of such simulations. The overlap between Eco and Pmi is significantly smaller than chance, and the small size of the overlap between Eco and Vch is very close to being statistically significant. Overall, this analysis indicates that the sizes of the intersections are substantially smaller than what would be expected by chance, and that the different Lrps are having significantly different effects on expression of the *E. coli *genome.

**Table 2:** Statistical analysis of permuted microarray data

**Pair**	**Size of overlap**	**# of overlaps with smaller size**	**P-value**
Eco and Pmi	273	16	0.04
Eco and Vch	226	21	0.052
Pmi and Vch	365	28	0.07

Second, we used intra-set comparisons to qualitatively evaluate the observed overlaps. We compared the fractions of genes, differentially expressed by both Lrps in a pairwise comparison, at different ratio cut-offs. We also compared *lrp vs*. WT (chromosomal *lrp*^+^), and *lrp*(P*araBAD*-LrpK12) *vs. lrp*. This allowed us to compare the overlaps between LrpK12 on two plasmids and between a plasmid and the WT chromosomal gene. The overlap fraction was calculated as the number of regulated genes in both of two sets, divided by the total number of unique genes in both sets. Fig. 6B illustrates that, as expected, the overlaps are consistently greater between identical complementation sets than between heterologous sets. Thus the microarray results reveal statistically significant functional differences between the Lrp orthologs.

### Microarray analysis of gene regulation by heterologous Lrp proteins – effects on magnitude of regulation

**Figure 7:**
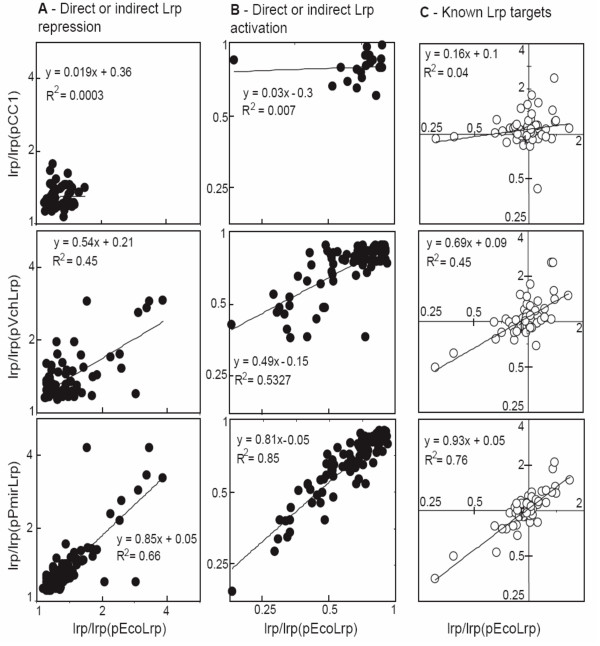
**Extent of regulatory conservation for significantly increased or decreased targets.** In every panel, the x-axis shows the gene expression ratio for *E. coli K-*12 Δ*lrp *relative to that in the same strain complemented by *E. coli lrp *(pEcoLrp). The y-axes indicate the equivalent ratio, where the complementation is by vector alone (pCC1) or the *lrp *alleles from *V. cholerae *(pVchLrp) or *P. mirabilis *(pPmiLrp). Full complementation relative to that by pEcoLrp would yield a slope of 1.0 for the linear fit. **A**. This column shows responses of the gene set yielding statistically-significant increases in expression associated with *lrp *mutation in *E. coli*, as reflected by an expression ratio significantly above 1.0 on the x-axis. This set includes genes that are repressed (directly or indirectly) by *E. coli *Lrp. **B**. This column includes the set of genes showing significant decreases in expression associated with *lrp *mutation in *E. coli*, indicating direct or indirect activation by *E. coli *Lrp. **C**. This column shows the set of 57 genes recognized in RegulonDB [71, 72] as being directly controlled by Lrp, whether the control is positive or negative. This set includes genes that are controlled by Lrp, but not under the growth conditions used by us, so the cluster of genes showing little or no effect of Lrp is not surprising [66, 69]. The relative transcript abundances were estimated from at least three independent biological replicas using a linear model similar to one introduced before [141, 142]. Significantly expressed genes were identified at a fixed false discovery rate of 5% at the 90^th ^percentile [138]. Details of the statistical analysis of these data are in the text, and a list of the 57 RegulonDB Lrp targets is in the Methods section.

Predictions of regulatory connections between a regulator and a target gene are useful in themselves, but substantial understanding of a cell's gene regulation also requires knowing the sign and strength of the regulation. Accordingly, we next examined whether the subset of genes that *was *regulated by both *E. coli *Lrp and one of the other orthologs showed a similar *magnitude *of regulation by each ortholog (Fig. 7). The effects of *E. coli lrp *(pEcoLrp) on gene expression are shown on the x-axis in every panel. Column A shows the set of genes for which transcript levels gave statistically significant decreases when the *lrp *mutation was complemented. Column A thus represents genes that are repressed (directly or indirectly) by *E. coli *Lrp. Column B includes gene showing direct or indirect activation by *E. coli *Lrp. Column C shows the set of 57 genes recognized in RegulonDB [71,72] as being directly controlled by Lrp, whether the control is positive or negative. This set includes genes that are only controlled by Lrp under growth conditions that differ from those used by us [66,69], so the cluster of genes showing little or no effect of Lrp is not surprising.

We used the slope of a least-squares fit between two ratios as a measure of overall regulatory concordance (Fig. 7). One ratio is the expression level of genes in the *E. coli *Δ*lrp *mutant over the level in that strain complemented by a plasmid carrying the *E. coli lrp *gene (on the x-axis in all panels). The second ratio (y-axis) is the expression in *E. coli *Δ*lrp *over that in the same strain complemented by a test plasmid (vector control, or *lrp *from *P. mirabilis *or *V. cholerae*). Full complementation relative to that by pEcoLrp would yield a slope of 1.0 for the linear fit. For each column, the top row indicates the effects of "complementing" the Δ*lrp *allele with the vector alone (pCC1). Not surprisingly, the negative control of "complementing" the *lrp *mutation with the vector gives slopes of <0.2 for all three gene sets. As a positive control, we carried out a similar analysis comparing on the one hand the *E. coli lrp *mutant to the mutant complemented with plasmid-borne *E. coli lrp *[*i.e*., *lrp*/*lrp*(pLrpK12)], and on the other hand the mutant to the WT *lrp*^+ ^strain (*lrp*/WT). The resulting slope between these datasets was within error of 1.0 (0.94 ± 0.12; not shown), indicating that native Lrp – even with the heterologous promoter and translation initiator – fully complements the effect of the chromosomal *lrp *mutation under the growth conditions used.

Supplying the *lrp *allele from *P. mirabilis *(pPmiLrp, bottom row) gave substantial, though not full, complementation of the gene expression pattern, with slopes ranging from 0.81 to 0.93. The *lrp *allele from *V. cholerae *(pVchLrp), which is more divergent from that of *E. coli *than is that of *P. mirabilis *(Fig. 1), yielded lower overall regulatory complementation – the slopes range from 0.49 to 0.69. Thus small changes in the Lrp sequence, outside of the fully-conserved helix-turn-helix motif, have substantial effects on the magnitude of Lrp-dependent effects on gene expression.

We find that, if anything, this analysis underestimates the differences between effects of the different Lrp orthologs. Since the power of inference cannot be adequately estimated in microarray experiments, we fixed the false discovery rate (FDR) at 5% without setting a minimum limit on fold change. However when we added a fold-change limit to the fixed FDR the differences between regression slopes were increased (not shown). In these experiments, genes were assigned to columns A or B (Fig. 7) based on having significant responses (positive or negative) to *E. coli *Lrp. The measurements on which this selection was based include noise, so even without a real difference in Lrp function the data might regress back towards no change in the complementation experiments. However, since the correlations used only genes that were significantly affected in the same direction in both sets in each pair, no systematic degradation of the signal is expected.

We evaluated the significance of differences between the slopes in Fig. 7 as follows. To avoid making assumptions about the nature of the distribution under the NULL hypothesis, we bootstrapped the slopes and estimated 95% confidence bands for each slope. The coordinates of each point in the correlations are estimates of corresponding ratios of transcript abundances, obtained as means of ratios observed in independent biological experiments. Therefore, we estimated the width of slopes of the regression lines by permuting the series, choosing N points (corresponding to the number of genes used in the correlation) at random from one of the three ratios. For example, for gene 1 we can take the ratio from biological replicate 1, for gene 2 from replicate 3, *etc*. N-member series were permuted in this way 1000 times, and the spread of 95% confidence intervals around the mean was calculated relative to the regression coefficients from Fig. 7. We demonstrated that 95% confidence intervals do not overlap between regression lines of interest. This supports the conclusion that differences between Lrp orthologs can explain the different amounts of variance observed in the various complemented strains.

### Levels of Lrp protein in *E. coli*, *P. mirabilis*, and *V. cholerae *in different media and growth phases

If orthologous regulatory proteins are to generate the same expression patterns for orthologous target genes, having the same *intrinsic *properties (DNA binding specificity and equivalent interactions with small molecules and with other proteins) would be neccesary but not sufficient. The orthologs would also have to share *extrinsic *properties, including accumulation to similar levels under various growth conditions. This provides the basis for a fourth test of implicit assumptions underlying regulatory extrapolation. Potential differences in Lrp levels were minimized, in the complementation experiments described above, by providing each *lrp *ortholog with a common promoter and translation initiation region. However *E. coli *growing exponentially in a minimal glucose medium accumulates three- to four-fold more Lrp than in rich medium [85,86]. To determine whether this pattern of Lrp accumulation is conserved, we used western blot analysis to measure the levels of Lrp throughout a batch growth cycle in *E. coli, P. mirabilis *and *V. cholerae *grown in MOPS glucose and MOPS defined rich media (supplemented as described in Methods).

**Figure 8:**
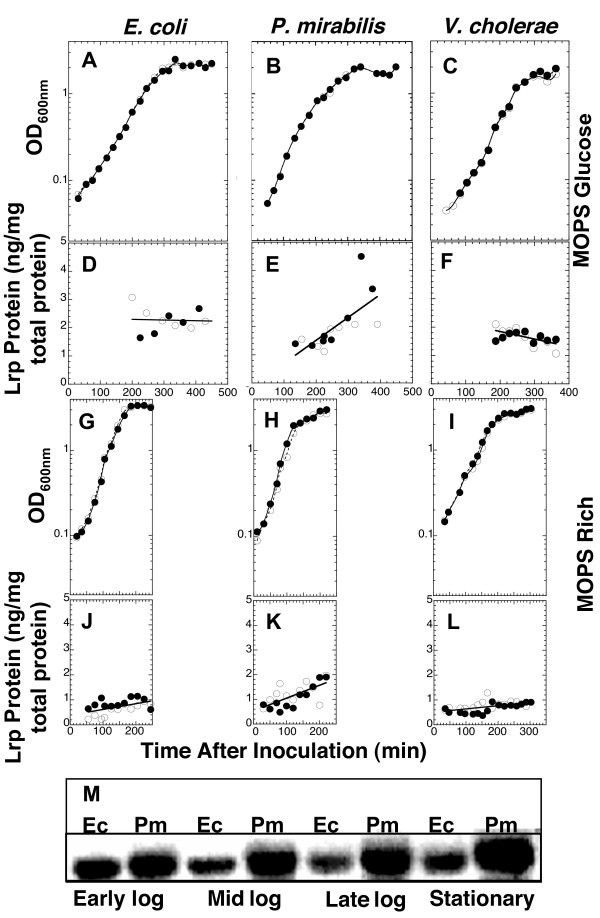
**Lrp protein levels as a function of growth.** Wild-type strains of *E. coli*, *P. mirabilis *and *V. cholerae *were grown in MOPS glucose plus nicotinate or MOPS glucose defined-rich media. The data are from two independent experiments (open and closed symbols). Growth curves (**A-C**, MOPS glucose medium; **G-I**, MOPS rich medium) and Lrp protein levels (**D-F**, glucose; **J-L**, rich) are shown. Equal amounts of total protein were loaded in each lane, and a standard curve of purified *E. coli *Lrp was included on each gel for quantitation. **M **shows a comparative western blot. Cell pellets were boiled and equal amounts of total protein from *E. coli *(Ec) and *P. mirabilis *(Pm) were resolved side-by-side via SDS polyacrylamide gel electrophoresis. The subsequent blot was probed with polyclonal antiserum raised against *E. coli *Lrp.

Our results confirm the earlier studies of *E. coli*, in that we saw several-fold higher Lrp levels when cells were grown in MOPS glucose than when they were grown in MOPS rich medium (Fig. 8, compare panels D and J). When grown in MOPS glucose, *P. mirabilis *(Fig. 8E) and *V. cholerae *(Fig. 8F) produced levels of Lrp similar to that in *E. coli *(Fig. 8D). Furthermore, all three species showed severalfold lower Lrp levels in rich than in minimal medium. There was, however, one substantial difference among the cultures. In the rich medium, *P. mirabilis *(Fig. 8K) produced up to twice as much Lrp as *E. coli *or *V. cholerae*, with levels highest in stationary phase.

These differences between *E. coli *and *P. mirabilis*, in Lrp protein levels, could well have substantial regulatory significance but needed confirmation. Samples were taken from parallel cultures of *E. coli *and *P. mirabilis *during early logarithmic, mid logarithmic, late logarithmic and stationary phases in defined rich medium. Equal amounts of total protein were resolved side-by-side via SDS PAGE. The results of the subsequent western blot (Fig. 8M) confirm that *P. mirabilis *produces substantially more Lrp protein throughout the growth phases, with the greatest difference (roughly twofold) seen in stationary phase. Thus Lrp provides an example of related bacteria with nearly-identical regulator proteins producing significantly different amounts of those regulators.

### Levels of *lrp *mRNA in *E. coli*, *P. mirabilis*, and *V. cholerae *in different media and growth phases

**Figure 9:**
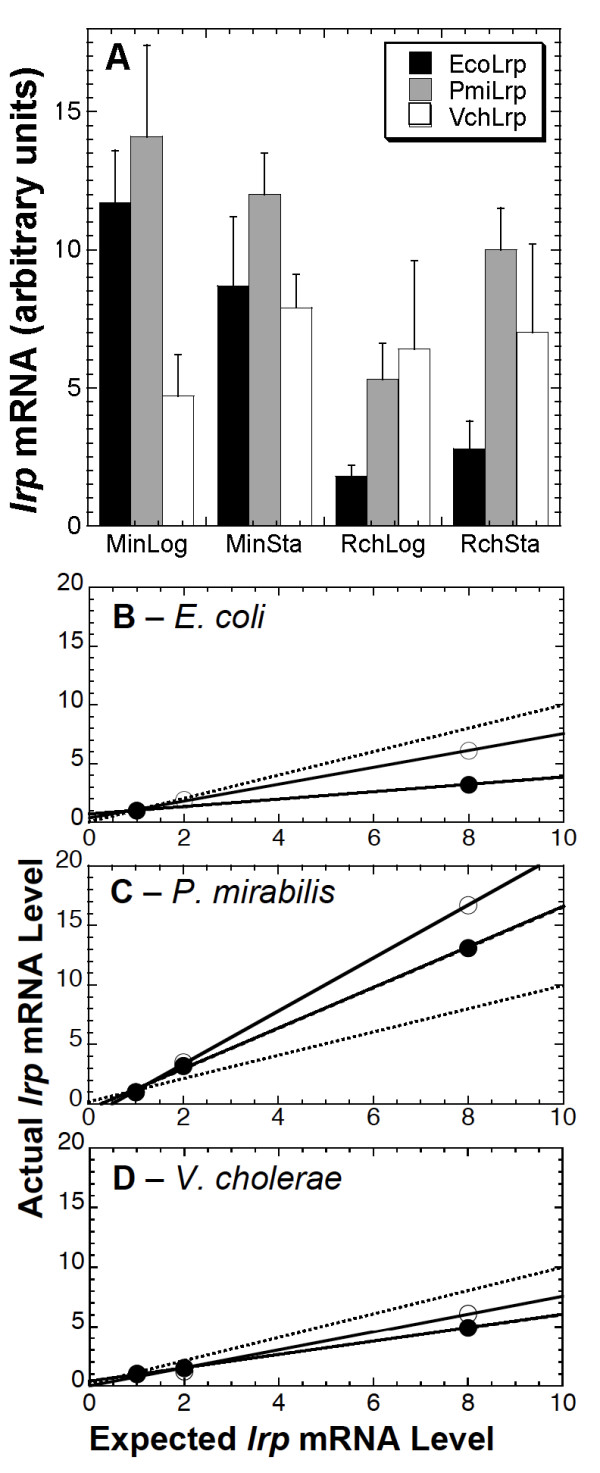
**Variation of *lrp *mRNA levels with growth phase and medium.A**. Values shown are arbitrary units from standard curve-based QRT-PCR (see Methods), with bars indicating standard errors. At least four points from each of two independent experiments were used to generate each plotted value. Conditions were MOPS-glucose minimal medium, supplemented as described in Methods, in logarithmic (MinLog) or stationary phase (MinSta), or MOPS glucose defined rich medium in those growth phases (RchLog, RchSta). **B-D**. Direct comparison of *lrp *mRNA levels.**B**. A baseline amount of total *E. coli *RNA (from a mid-log phase culture in MOPS glucose plus nicotinate) was mixed with varying amounts of test RNA (all from log-phase cultures) from glucose (open circle) or rich (closed circle) cultures. The mixes were used as template for simultaneous amplification with three primer pairs. If the test cDNA preparation has the same proportion of *lrp *cDNA as the reference pool, the detected amount of *lrp *cDNA should rise with a slope of 1.0 (actual *vs*. detected, based on the varied amounts of test cDNA added); this is shown as a dotted line in each panel.

As regulator levels are often inferred from microarray measurements of mRNA levels, we determined whether the level of *lrp *mRNA also varies with the growth medium in all three organisms (Fig. 9A). At least four QRT-PCR determinations from each of two independent experiments were averaged to generate each plotted value. In *E. coli*, *lrp *mRNA levels are profoundly lower in rich than in minimal medium, irrespective of growth phase, and similar to what was seen for Lrp protein (compare black bars to Fig. 8 panels D and J). Also like the protein results, *P. mirabilis lrp *mRNA rises in stationary phase (compare gray bars to Fig. 8 panels E and K), but the mRNA shows this rise only in rich medium. The *V. cholerae lrp *mRNA results resemble the protein data in that there is no significant growth phase effect, but differ in that the mRNA shows no growth medium effect (compare white bars to Fig. 8 panels F and L).

We also measured the levels of *lrp *mRNA for all three organisms during log-phase growth, using a more highly-quantitative method, and the results are consistent with the protein data (Fig. 9B–D). We used a sensitive dilution-response approach that makes use of the fact that our three species-specific pairs of QRT-PCR primers for *lrp *amplify with the same efficiency but are completely specific for their respective template DNAs (data not shown). A standard amount of reference *E. coli *RNA (from a mid-log phase culture in MOPS glucose plus nicotinate) was mixed with varying amounts of test RNA (from cultures grown in either the MOPS glucose or MOPS rich medium). The various mixes were reverse transcribed and the resultant cDNA was used as template for simultaneous amplification with the three primer pairs, with real-time fluorescence monitoring (see Methods). Where the proportion of total mRNA as *lrp *mRNA is equal to that in the reference sample, the slope should be 1.0 (dotted lines in Fig. 9B–D). For *E. coli-*derived cDNA, the resulting slope is about 0.75 (MOPS glucose culture; Fig. 9B) or 0.4 (MOPS rich culture), indicating lower *lrp *mRNA levels in rich than in minimal medium and consistent with the medium-dependent effect on Lrp protein levels shown in Fig. 8. *V. cholerae *(Fig. 9D) gave a pattern similar to that of *E. coli*, though with less of a medium-dependent effect. However – also consistent with the protein data – *P. mirabilis *had substantially more *lrp *cDNA as a proportion of total cDNA than did *E. coli*, with slopes of about 2 (Fig. 9C).

### Regulation of orthologous target genes in their native backgrounds

**Figure 10:**
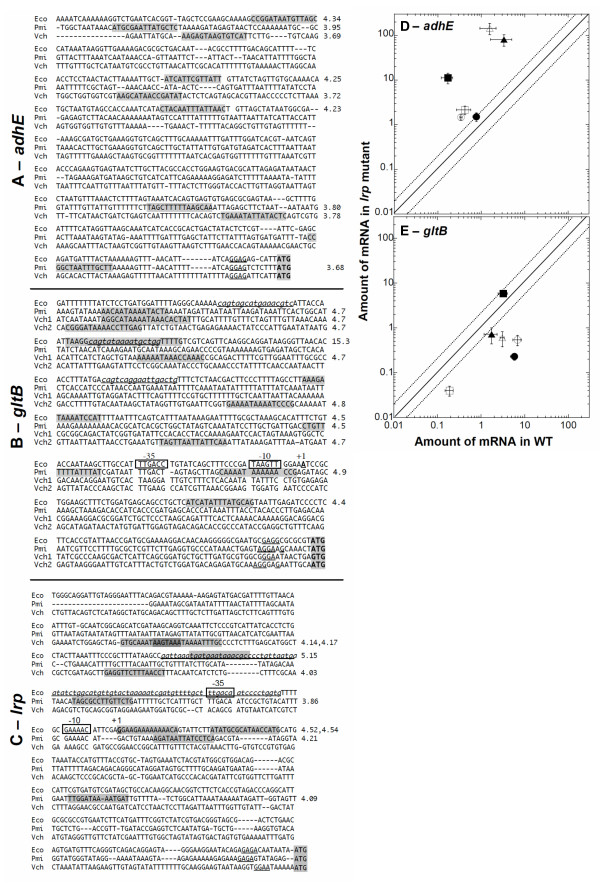
Regulation of orthologous target genes in native backgrounds. **A-C: **Sequences upstream of *adhE*, *gltB *and *lrp *orthologs. In each case, the sequence ends with the initiation codon. Lrp-binding sites and the transcriptional +1 position are known for *E. coli K*-12 [112]. Demonstrated Lrp binding sites are in *underlined lowercase italics*, and the -35 and -10 sequences inferred from the known +1 position (for *E. coli*) are boxed. Putative binding sites, predicted by the PRODORIC virtual footprinter [102] are shaded, and the match scores for predicted sites are shown to the right. For *E. coli *P*gltB*, one of the predicted sites overlaps an actual site, and gives a particularly high match score, though an overlapping actual site in P*lrp *does not. *V. cholerae *has two nearly-tandem copies of the *gltBD *operon on chromosome I. The 5'-most *gltB *isozyme ("Vch1", locus tag Vc2373) is 43% identical to Eco *gltB*, while the 3'-most isozyme ("Vch2", Vc2376) is 73% identical to Eco *gltB *in amino acid sequence. **D-E: **Samples were isolated at an OD_600 nm _of 0.3 (log), as well as 1 h after linear growth stopped (stationary), from *E. coli, P. mirabilis *and *V. cholerae *wild-type and *lrp *cultures growing in MOPS defined rich medium. QRT-PCR was used to determine the relative levels of *adhE, gltB *and *recA *messages, with *recA *serving to provide a Lrp-independent baseline. The experiment was performed in triplicate and the level of message was determined using the standard curve method and normalization to *recA*. **D **– *adhE*. **E **– *gltB*. For each plot filled symbols represent log phase levels and open symbols represent stationary phase levels. The symbol shapes indicate the species: *P. mirabilis *(triangles) *E. coli *(circles) or *V. cholerae *(Vc2373, squares). The line indicates the position for data if no effect of Lrp is seen (ratio of 1); points above the line are consistent with repression, while those below the line are consistent with activation by Lrp. The dotted lines show, to facilitate comparison, the borders of a twofold effect.

We next tested an explicit assumption of regulatory extrapolation, by determining if the expression of orthologs of *E. coli *Lrp target genes are Lrp-responsive in their native hosts. We measured the mRNA levels from orthologs of two genes previously shown to be Lrp responsive in *E. coli *[69]: *adhE *and *gltB*. These orthologs were chosen based on percent identity to the *E. coli *protein, and presence of at least one predicted Lrp-binding site using PRODORIC [102] (Fig. 10).

Wild-type and *lrp *strain pairs of *E. coli*, *P. mirabilis*, and *V. cholerae *were grown in MOPS glucose defined rich medium. Samples were taken in early logarithmic phase (OD_600 nm _= 0.3), and early stationary phase (1 h after the culture OD *vs*. time semilogarithmic plot diverged from linearity). Real-time RT-PCR analysis was used to determine the relative levels of *adhE*, *gltB*, and (as a control) *recA *mRNAs. The experiment was performed in triplicate and the relative levels of mRNA were determined using the standard curve method [103] and by normalizing to *recA*. There is no effect of Lrp on *recA *expression, at least in *E. coli *and *V. cholerae *under our conditions [69] and N. Dolganov, pers. commun.).

#### Regulation of *adhE*

AdhE is a fused acetaldehyde-CoA dehydrogenase, iron-dependent alcohol dehydrogenase and pyruvate-formate lyase deactivase [104-106]. In *E. coli*, *adhE *is preferentially expressed in stationary phase [107-109], and repressed by Lrp in a leucine-independent manner during exponential growth in minimal glucose medium [69] and ABK, unpublished data). Fig. 10D is a log-scale correlogram showing the regulatory pattern of *adhE *in all three organisms. If Lrp had no effect on *adhE *mRNA levels, then the points would fall on the diagonal line. The fact that all points are above the diagonal line is consistent with Lrp-dependent repression in all three species. However a more detailed analysis of this data reveals that the regulatory patterns from *P. mirabilis *and *V. cholerae *are different from those in *E. coli *and from one another. In *E. coli *(circles), there was a modest Lrp-dependent decrease in *adhE *mRNA in log phase. *P. mirabilis*, in contrast (triangles), showed a strong repressive effect of Lrp, though no real growth-phase dependent change in expression. *V. cholerae *(squares) exhibited slight Lrp-associated reduction in log phase expression, but in stationary phase the *adhE *mRNA levels were about 50-fold higher in the *lrp *mutant strain.

#### Regulation of *gltB*

The other target gene, *gltB*, was described earlier. *V. cholerae *appears to have two tandem *gltB *isozyme genes, with 73% and 43% amino acid identity to *E. coli *GltB. The presence of all conserved domains and key residues strongly suggests that both of these genes actually specify GltB [110,111] and M.A. Vanoni, pers. commun.). We failed to detect expression of the Vibrio *gltB *with higher identity to *E. coli *during growth in minimal glucose and defined rich medium (Vc2376, not shown), however the lower-identity isozyme (Vc2373) was expressed. In *E. coli gltB *is activated 30–40 fold by Lrp when grown in MOPS glucose [67,69,74,92], with the activation codependent on another global regulator, IHF [93,94]. We have already shown that the Lrp orthologs from *P. mirabilis *and *V. cholerae *effectively replace *E. coli *Lrp, in an *E. coli *background, for activation of *E. coli *P*gltB *(Fig. 4, panels C and F). Here we determine whether the *Proteus *and *Vibrio *Lrp orthologs each activate their native *gltB *promoters in the native background. The *P. mirabilis lrp *strain did not grow well in the MOPS glucose medium used in this study, so all experiments were carried out in MOPS rich medium. In another rich medium (LB), activation of *gltB *by Lrp is reduced relative to minimal glucose, but is still about triple the level in a *lrp *disruptant [67].

We found that in *E. coli gltB *is activated ~25 fold by Lrp during mid-log, and about half as much in early stationary phase (Fig. 10E, circles). In *P. mirabilis *there was several-fold more log-phase *gltB *expression in the *lrp*^+ ^than in the *lrp *strain, with little if any growth-phase-dependent change. *V. cholerae *gave the most divergent expression pattern: *gltB *mRNA levels were halved by Lrp in log phase, but increased about fivefold by Lrp in early stationary phase. Bearing in mind that this is the ortholog showing only 43% identity to *E. coli gltB*, it is nevertheless the case that while Lrp activates *gltB *in log-phase *E. coli *and *P. mirabilis*, under the same conditions it slightly represses Vc2373 in *V. cholerae*.

### Lrp regulatory interactions with two promoter regions

Finally we tested whether promoter regions from orthologous genes, where the *E. coli *gene is Lrp-controlled, are regulated by Lrp in heterologous hosts. This was done by preparing *lacZ *operon fusions to a set of ortholog promoters cloned from *E. coli*, *P. mirabilis*, and *V. cholerae*, and then introducing each of these fusions into both the WT and *lrp *strains of all three species. Relative LacZ activity was measured using the approach shown in Fig. 4 (determining the slope of a LacZ activity *vs*. culture density plot). These experiments are reciprocal to those shown in Fig. 4, where heterologous *lrp *alleles had been moved into an *E. coli *background to test for control of *E. coli *target genes. However the two sets of experiments share the feature that the target promoters/binding sites being assessed are identical (the set of *E. coli *promoters in the case of Fig. 4, and the promoter for one of the three *lrp *orthologs in this experiment).

#### Regulation of P*lrp*

**Figure 11:**
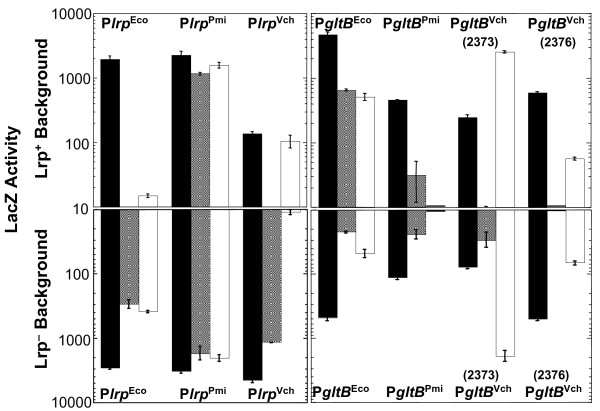
**Lrp effects on orthologous promoter regions in three backgrounds.** The orthologous P*lrp *or P*gltB *regions were amplified from *E. coli*, *P. mirabilis*, and *V. cholerae *and inserted upstream of a promoterless *lacZ *gene. These plasmids were then used to transform *lrp*/*lrp*^+ ^strain pairs of all three species, and *lacZ *was measured *vs*. culture density to obtain the slopes. Black bars indicate expression in the *E. coli *WT (upper panels) or *lrp *(lower panels) background, gray bars indicate expression in the *P. mirabilis *strain pair, and white bars represent expression in the *V. cholerae *strain pair. For each least squares fit, yielding the plotted slope value, the correlation coefficient was ≥0.97. The standard error for each slope was calculated from the residuals using the "summary(lm(y~x))" function from the R statistical package. All strains were grown in MOPS glucose medium supplemented with nicotinate, pantothenate, thiamine, methionine and cysteine (see Methods).

One promoter set was P*lrp *from *E. coli*, *P. mirabilis*, or *V. cholerae*. P*lrp *in *E. coli *is autogenously repressed [89], and all three *lrp *promoters in all three hosts (with one exception) show lower expression in the presence of Lrp than in its absence (Fig. 11). However, the exception is the *V. cholerae *P*lrp *in its native host, which shows tenfold *higher *expression in the presence of Lrp, suggesting activation rather than repression. As the same plasmid, but with different *lrp *promoters upstream of *lacZ*, gives a repression phenotype in the *V. cholerae *background, this P*lrp *activation result is unlikely to result from copy number variations in the vector.

Aside from this, there is considerable variation in the level of repression. *E. coli *P*lrp *is much more strongly repressed by Lrp in the heterologous hosts than in the native host. P*lrp *of *P. mirabilis*, unlike that from the other two organisms, shows no evidence of autogenous repression by Lrp – *P. mirabilis *P*lrp *is only weakly repressed (1.3–1.4-fold) in all three hosts. This may explain the higher levels of Lrp we found in this organism (Figs. 7 and 8), and raises interesting questions about how Lrp levels are controlled in *P. mirabilis*. In summary, both the source of the promoter and the host background strongly affected the regulatory pattern.

#### Regulation of P*gltB*

The second promoter analyzed in this way was P*gltB*, including the promoters from both *V. cholerae *putative isozymes. In *E. coli*, P*gltB *is activated by Lrp [74,92-94,112], but the results in Fig. 11 are more varied than for P*lrp*. *E. coli *P*gltB *was the only one to be consistently expressed and activated by Lrp (10–30-fold) in all three hosts. P*gltB *from *P. mirabilis *was barely expressed in *V. cholerae *irrespective of Lrp, was expressed weakly in its native host (under the conditions used) and with statistically insignificant effects of Lrp, while in *E. coli *this promoter was expressed at a moderate level and was activated by Lrp (5-fold). The *V. cholerae *P*gltB *that is less similar to *E. coli gltB *(Vc2373) was strongly expressed in its native host, with no significant effect of Lrp, while showing lower expression levels and activation (3-fold) in *E. coli*, and yielding low expression but repression by Lrp (3-fold) in the *P. mirabilis *background. The other *V. cholerae *P*gltB *(Vc2376), more closely related to *E. coli gltB *of the two, had given undetectible expression in our RT-PCR assays. Vc2376 gave detectable expression in the form of a *lacZ *fusion, though as with the RT-PCR analysis it yielded much lower expression in the native host than did Vc2373. Lrp had insignificant effects on Vc2376 in the native host and in *E. coli*, while in *P. mirabilis *there was very low expression irrespective of Lrp. Thus, as with P*lrp*, the promoter behavior varied substantially between hosts with respect to Lrp effects, and given hosts expressed the promoters of orthologous target genes in varied manners.

Conserved regulation of P*lrp *and P*gltB *would have led to greater similarity of expression patterns of the three (or four) orthologs in the three host species. However, the differences may reflect more than just differences in Lrp. In *E. coli*, P*gltB *is controlled by Lrp, Crp, IHF, and ArgR [67,74,93,94,112]. Species or promoter binding-site differences affecting the other regulators (besides Lrp) could contribute to the different observed behaviors – in an extreme case, Lrp has no effect on P*gltB *in the absence of IHF binding [93]. A similar caveat may apply to P*lrp*, which in addition to being autogenously repressed by Lrp [89] may be activated by GadE in *E. coli *[113].

## Discussion

Robust methods for predicting gene regulation from DNA sequence data would greatly increase the usefulness of the rapidly-expanding collection of bacterial genome sequences. However current methods rely on a hypothesis that has received limited testing – that a well-conserved regulator, and well-conserved target gene downstream of a putative binding site for the regulator, together imply a similar pattern of regulation (or at least *some *direct regulation). For brevity, we refer to this as the "regulatory extrapolation hypothesis," since it involves inference of a regulatory pattern based on conservation with respect to a well-studied reference organism. Some possibilities regarding this hypothesis are: that it is generally true among closely-related organisms (genetically, ecologically, or both), that it is generally true for only the most-highly conserved regulators and target genes, or that it is often incorrect even among highly-related genes and organisms. We have studied regulatory extrapolation by examining a well-conserved global regulator (Lrp), conserved genes that are Lrp regulatory targets in *E. coli*, and two species of increasing but limited genetic distance from *E. coli*: *Proteus mirabilis *and *Vibrio cholerae*.

### Closely-related Lrp proteins have significant intrinsic differences

Regulatory extrapolation relies on a tacit assumption that regulatory proteins with high amino sequence identity are functionally equivalent. We took closely-related Lrp orthologs (all >92% identity) from three species, gave them identical expression sequences for transcription and translation, put them into the same very low-copy vector (pCC1), and introduced them into the same *E. coli K*-12 *lrp *and *P. mirabilis lrp *backgrounds. Of the 164 aa in all three Lrp orthologs, *P. mirabilis *Lrp differs from *E. coli *Lrp at only four positions, while the *V. cholerae *and *E. coli *orthologs differ at 12; none of these differences affects the known DNA-binding helix-turn-helix or the coregulator binding sites (Fig.1).

The Lrps exhibited similar overall behavior, supporting extrapolation in general, particularly where the only concern is whether a regulatory link exists at all irrespective of its sign or strength. However there were significant functional distinctions between the tested Lrp orthologs. In *E. coli*, the native P*lrp *(fused to *lacZ*) was repressed equivalently by *E. coli *and *V. cholerae *Lrp, but about twice as much by *P. mirabilis *Lrp (Fig. 4B). These differences were magnified in the presence of leucine, where *P. mirabilis *Lrp was unique in showing virtually no effect (Fig. 4E). In contrast, the three Lrp orthologs gave equivalent activation of P*gltB *(Figs. 4C, F). P*livK*, which in *E. coli *is activated by Lrp in the absence of leucine and repressed in its presence, was regulated equally by all three Lrp orthologs with one exception: in the presence of leucine, the *E. coli *Lrp represses less than the others (Fig. 4D, G).

We used microarray analysis to more globally assess the ability of orthologous Lrp proteins to properly control the *E. coli K-*12 Lrp regulon. Our results confirmed that minor changes in the Lrp amino acid sequence had substantial effects on the targets (Fig. 5) and magnitude (Fig. 7) of Lrp effects. Of the genes whose expression was significantly changed by *E. coli *Lrp, over a third were not significantly affected by either of the other Lrp orthologs. Conversely, about half of the genes significantly affected by *P. mirabilis *Lrp, and about a third of those affected by *V. cholerae *Lrp, were also affected by *E. coli *Lrp in the *E. coli *background. Looking only at the subset of genes that *were *significantly affected by all three Lrps, we found that whether we examined genes significantly repressed by Lrp, activated by Lrp (in both cases including both direct and indirect effects), or directly controlled by Lrp in *E. coli*, the results were consistent. Specifically, the transcriptional effects of *P. mirabilis *Lrp were most similar to those of *E. coli *Lrp (correlation of 80–92%), followed by *V. cholerae *(correlation of 67–70%), with vector alone showing no statistically significant similarity (0–20%), and the chromosomal *vs*. plasmid-borne *E. coli lrp *comparison giving 94% concordance. It is important that in these experiments, the only variable is the Lrp ortholog present – the target genes and binding sites are identical between strains (as they are all based on the same *E. coli *host).

Another assessment of Lrp functionality involved *P. mirabilis *swarming over a solid surface. Swarming is a complex phenomenon; for example, in *Salmonella *about a third of all genes showed swarming-associated changes in expression [114]. For the purposes of the present study, swarming thus represents a sensitive indicator of Lrp action. In a *P. mirabilis *background, we found that all three Lrp orthologs restored swarming, but gave repeatable differences in the resulting swarming patterns (Fig. 3).

We are currently exploring the molecular bases for these intrinsic differences in the three Lrp orthologs. In theory, a combination of differences in DNA specificity (due to changes outside the helix-turn-helix motif), in cooperativity, in response to coregulatory amino acids, or to interactions with RNA polymerase or other regulatory proteins [115] could be involved. For the purposes of this report, however, the main point is simply that such differences exist even between proteins that are 98% identical (the *P. mirabilis *and *E. coli *Lrps).

### Orthologous Lrp proteins can have different extrinsic properties

We also examined the native regulation of *lrp *in *E. coli*, *P. mirabilis*, and *V. cholerae*. Regulatory extrapolation relies on a second tacit assumption: that levels of the conserved regulator are similar in the organisms being compared, and change similarly in response to growth conditions. Target gene regulation is, not surprisingly, affected by the level of the regulatory protein; this is specifically true for Lrp [66,92,116].

Lrp protein levels in all three species were reduced in rich medium relative to glucose minimal medium (Fig. 8). For two of the three species, *lrp *mRNA levels are also lower in rich medium (Fig. 9). However we found that, during growth in defined rich medium (especially at higher cell densities), *P. mirabilis *levels of Lrp protein and *lrp *mRNA were about double those in *E. coli *or *V. cholerae *(Figs. 8, 9). For the purposes of this study, the important point is that Lrp levels differ significantly between the species, so that sequence analysis of the Lrp open reading frame and target gene promoter is not sufficient to predict expression patterns of the target gene. This regulatory variation is not an idiosyncracy of Lrp; for example, similar species-specific variation in regulation, among Enterobacteriaceae, has also been reported for the global regulator Fis [117], though as with Lrp the most basic patterns of expression are conserved [118].

### Orthologous target genes are regulated differently by the same Lrp protein

A third tacit assumption underlying regulatory extrapolation is the reciprocal of the one described immediately above: that orthologous target genes moved into a common background will be regulated in the same way by orthologous regulators. We prepared *lacZ *fusions to both P*gltB *and P*lrp *promoters from *E. coli*, *P. mirabilis*, and *V. cholerae*, and introduced them into the *lrp *and *lrp*^+ ^strain pairs for all three species in all combinations.

Once again the assumption is supported in general – most P*lrp *combinations are unaffected or repressed by Lrp (Fig. 11D), while all but one of the P*gltB *combinations are unaffected or activated by Lrp (Fig. 11E). [Note: repression and activation by Lrp have been demonstrated for these target genes in *E. coli*, but have not been proven to occur in the other backgrounds (where the effects might be indirect), and we use these terms for brevity.]

However the assumption is not supported by the specifics – *e.g*., the *E. coli *P*gltB *is well-expressed and Lrp-activated in all backgrounds, while the *P. mirabilis *P*gltB *ranges from nonexpression to Lrp-activated expression in the different backgrounds (Fig. 11). It is particularly interesting that, unique to the *V. cholerae *P*lrp *in the *V. cholerae *background, Lrp activated rather than repressing the promoter. There are some intriguing and distinctive sequence characteristics of the *V. cholerae *P*lrp *that may explain this behavior, and we are investigating these further. However *V. cholerae *P*lrp *gave low expression in the *V. cholerae *background even when Lrp was present, while this same promoter gave much higher (and Lrp-responsive) expression in the *E. coli *and *P. mirabilis *hosts. This, and the fact that the *E. coli *and *P. mirabilis *P*lrp *fusions were both well expressed in the Vibrio background, suggests that *V. cholerae *negatively regulates its P*lrp *via some *Vibrio*-specific factor, and that Lrp may in this case act as an anti-repressor.

Comparisons of independent microarray studies on distinct platforms are problematic [119,120], and this report has the benefit of direct comparison using the same experimental and statistical methods. Nonetheless, our results are supported by earlier analyses of the somewhat differing effects of gene disruptions in the global regulators H-NS [121-124], IHF [125,126], and Fis [127,128] in the closely-related genera Escherichia and Salmonella.

One possible interpretation of these results is that global regulators are more likely to have greater recognition plasticity than local regulators, in which case a study of Lrp may represent something of a "worst case scenario" for regulatory extrapolation despite its remarkable conservation (Figs. 1, S1). It has been suggested that global regulators bind a large number of sites with a wide range of affinities, affecting chromosome superhelical density and providing a continuous "analog" regulation, in contrast to the more "digital" regulation by more specific regulators [129]. It is also true, however, that more local and sequence-specific regulators (such as LexA) show considerable range in their *in vivo *DNA binding [130]. There are certainly local regulators that bind unique sites with extreme specificity [131], though the value of predicting their regulatory roles across species is correspondingly limited.

## Conclusion

Our results present a mixed picture. In general terms, we found that Lrp behaves in similar ways in the three tested species. However we also found significant intrinsic and extrinsic differences among the Lrp orthologs, and differences in the behavior of target gene promoters having predicted Lrp-binding sites, despite the fairly close genetic relatedness of the species we examined. These results suggest that regulatory extrapolation over limited genetic distances, with the goal of making fairly general predictions of regulon structures, can provide valid and useful insights. However our results also indicate that the strength and sign (positive or negative) of the regulation, even across limited genetic distances, is surprisingly variable.

## Methods

### Bacterial strains, media, and growth conditions

The bacterial strains used for this study are listed in Table 1. In all cases cells were grown in baffled flasks shaken at 37°C. Morpholinopropane sulfonic acid (MOPS) glucose minimal medium, and MOPS-based defined rich medium [132] were purchased from Teknova (Hollister, CA). In experiments comparing *E. coli *and *V. cholerae *with *P. mirabilis*, media for all strains were supplemented with 0.01 mM nicotinic acid, which is required for the growth of *Proteus mirabilis *[56] and of the *lrp *mutant of *Vibrio cholerae *(REL, unpublished observation). When *lrp *mutants were part of an experiment, minimal media also contained 0.01 mM each of pantothenate and thiamine, which we found to be additional requirements of the *P. mirabilis lrp *mutant, and in some cases 0.1 mM L-cysteine with 0.2 mM L-methionine which were not required but improved growth of this mutant (REL, unpublished observation). For P*livK-lacZ *analyses, additional amino acids were used at the following final concentrations: 10 mM L-leucine, 0.4 mM L-isoleucine and 0.4 mM L-valine. Antibiotics were used, where indicated, as follows: 100 μg ampicillin/ml, 15 μg chloramphenicol/ml, 100 μg kanamycin/ml, and 10 μg tetracycline/ml.

The *lrp *alleles are as follows. For *E. coli *and *V. cholerae*, all but the first six and last six codons of the *lrp *ORF were replaced by the gene for chloramphenicol acetyltransferase (*cat*) (our unpublished result; N. Dolganov and G. Schoolnik, unpublished result), with confirmation by PCR amplification and sequencing. Some experiments made use of strains carrying an *E. coli lrp::Tn*10 allele (*lrp-35*, [67]). The *P. mirabilis *allele is a *lrp::*mini*Tn*5 disruptant [84], provided by G. Fraser). The *E. coli *MG1655 *lrp *mutant has the entire *lrp *ORF replaced by the gene for kanamycin, and was constructed using λ*red *recombinase gene replacement system [133]. Other strain information is in Table 1.

### Growth experiments and sample isolation

Overnight cultures in MOPS glucose or MOPS rich medium were inoculated from (respectively) M9 glucose or LB agar plates containing 0.01 mM nicotinic acid, and grown to early stationary phase. These cultures were then used to inoculate fresh media (1:32). OD_600 nm _was measured following sample dilution as needed to maintain OD within the range of 0.08–0.3.

Samples for real-time RT-PCR analysis were isolated at the indicated times by removing an equal number of cells (estimated from culture density) from the flask and immediately adding it to two volumes of RNA stabilization buffer (RNA Protect Bacteria Reagent, Qiagen, Valencia, CA). This prevents the rapid changes in mRNA content that otherwise occur when bacteria are harvested. Samples were mixed, left at room temperature for 10 min, and stored at 4°C for no more than 5 days.

Samples for microarray analysis were isolated at an OD_600 _of ~0.4, at which point 20 ml of culture was mixed with 2.5 ml of ice-cold 5% water-saturated phenol (pH < 7.0) in ethanol [134]. After 10 min on ice, cells were pelleted, supernatant was removed, and pellets were frozen in liquid nitrogen and stored at -80°C, if necessary.

### RNA Isolation and cDNA synthesis

For RT-PCR experiments total RNA was isolated using the RNeasy miniprep kit (Qiagen) using their protocol with an added sonication step. Briefly, cells in the stabilization buffer were harvested by centrifuging at 4°C for 15 min at 5,000 rpm. Supernatants were removed and the pellet was resuspended in 1× TE buffer containing lysozyme (400 μg/mL). Lysis buffer was added and the cells were sonicated 3× for 15 s in a cup horn attachment to enhance lysis. Following ethanol precipitation, RNA was bound to the column provided, washed and eluted. To eliminate DNA, the RNA was treated with RQ1 RNAse-free DNAse (Promega, Madison, WI) as directed. cDNA was synthesized using total RNA as template, random hexamers (Invitrogen, Carlsbad, CA), and ImPromII reverse transcriptase (Promega). The random primers were annealed at 25°C for 5 min, and the first strand was then extended at 42°C for 1 h. The reverse transcriptase was inactivated by heating to 70°C for 10 min. cDNA samples were stored at -20°C.

For microarray experiments total RNA was extracted by the hot phenol-chloroform method [135], and treated with DNase I in the presence of RNase inhibitor for subsequent labelling by reverse transcription with Cy3-dUTP and Cy5-dUTP fluorescent dyes (Amersham, Little Chalfont, United Kingdom). The RNeasy miniprep kit (see above) was also used in some cases.

### Real time RT-PCR analysis

Primer sets (Integrated DNA Technologies, Coralville, IA) were designed for the *adhE, gltB, lrp *and *recA *genes for each strain (Table S1). Before each new experiment dilutions of cDNAs were tested to determine the concentration that gave maximally-efficient amplification, and to determine the efficiency for each primer set (23). Cycle threshold (C_T_) values were determined by Roche Lightcycler detection of SYBR green fluorescence. Melting curve (Roche Lightcycler software) and agarose gel analyses were used to confirm the formation of specific products, which ranged in size from 192–202 bp. The standard curve method was used to determine relative amounts of mRNA and levels were normalized to *recA *[103].

### Dilution-response RT-PCR

RNA was extracted from mid-log cells grown in MOPS glucose or MOPS rich media (plus nicotinate). RNA from the various samples was quantitated spectrophotometrically, diluted such that all samples had the same total RNA concentration, and then mixed 1:0, 1:1 and 1:7 with a standard level of RNA taken from *E. coli *grown in MOPS glucose (*e.g*., 1 μl *E. coli *MOPS glucose RNA mixed with 0, 1, or 7 μl *P. mirabilis *RNA). The RNA mixtures were then reverse transcribed (see above), and RT-PCR was performed using a 1:1:1 mixture of the *lrp *primer sets specific to each organism. Actual C_T _values were then plotted against the C_T _values expected if all original samples had the same proportion of *lrp *mRNA to total RNA. The resulting slopes indicate the fraction of *lrp-*specific cDNA relative to that in the reference *E. coli *sample.

### Western blot analysis

For each sample equal volumes of cells were centrifuged at 16,000 – *g *for two min. The supernatants were removed and the cell pellets were stored at -80°C until analysis. Pellets were resuspended in 1× SDS buffer (Novagen, Madison, WI). Cells were lysed by heating to 98°C for ten min, and total protein concentrations were determined using the RC/DC kit and protocol (BioRad, Hercules, CA). Equal amounts of protein were loaded on a 12% acrylamide SDS gel and electrophoresed at 110 V in 1× tris-glycine buffer. Proteins were then electroblotted to polyvinylidene difluoride (PVDF) membranes at 30 V for 1 h using the Xcell blot apparatus (Invitrogen). Proteins were detected by fluorescence using the ECL-plus Western Blotting Detection System (GE Health Sciences, Piscataway, NJ) per the manufacturer's protocol, with a 1:125 dilution of rabbit anti-Lrp polyclonal serum (gift of Dr. Joseph Calvo [87]), and a 1:25,000 dilution of horseradish peroxidase-conjugated goat anti-rabbit IgG (gift of Dr. Darren Sledjeski). Protein bands were visualized on a Storm 840I phosphorimager (Molecular Dynamics, now GE Healthcare). Densitometric analysis of Lrp bands was performed using the Molecular Dynamics software, and the amount of Lrp in each sample was determined by comparison to a standard curve from purified Lrp dilutions included on each gel.

### Microarray experiments

Starting with freshly-streaked single colonies, cultures (supplemented with Ile, Leu, Val and thiamine as described above) were aerobically grown overnight at 37°C and then diluted 20-fold into 20 ml of fresh medium. Recombinant cultures were propagated in the presence of chloramphenicol, and growth was monitored via OD_600 nm_. Cultures were maintained in exponential growth for at least 10 generations by dilution.

Relative mRNA abundances between the *lrp *mutant and the same strain carrying a *lrp *gene on plasmid pCC1 (or carrying only the vector) were determined, using at least three biological replicates. Each replicate culture was grown on a different day, and inoculated with a mix of 2–3 average-sized colonies less than a day old. This analysis employed *E. coli K-*12 whole-genome DNA microarrays including 99% of all annotated open reading frames and the stable RNA genes. Slide preparation, reverse transcription with the Cy-dyes, hybridization, and image scanning were performed as previously described [135]. The fluorescent probes were hybridized to an array at 65°C for 6 h. Intensities in both channels were smoothed using the Lowess method [136]. Some biological replicate samples were split into technical replicates, on which dye-swap analyses were conducted. Known Lrp targets were taken from RegulonDB [71,72], and are listed below.

aidB aroA b2659 dadA dadX fimA fimC fimD fimE

fimF fimG fimH fimI gabP gabT gcvH gcvP gcvT

gltB gltD gltF ilvA ilvD ilvE ilvG_1 ilvG_2 ilvH

ilvI ilvL ilvM kbl livF livG livH livJ livK

livM lrp lysU malT micF ompC ompF oppA oppB

oppC oppD oppF osmC osmY sdaA serA serC stpA

tdh yeiL ygaF

### Statistical analysis of microarray data

Dye- and array-specific noise was removed using the analysis of variance (ANOVA) error model [137]. In pair-wise comparisons, differentially expressed genes were identified at an estimated false discovery rate of less than 5% using the two-class T-test in the SAM package [138]. The NULL hypothesis was that gene-specific intensities in two classes have indistinguishable means.

### β-galactosidase assays

Strains were grown to exponential phase in glucose minimal MOPS medium (Teknova). Samples were taken at 20 and 30-min intervals throughout the growth period. Levels of β-galactosidase were determined by *o*-nitrophenyl-β-D-galactoside (ONPG) hydrolysis [139]. β-galactosidase levels were plotted against culture absorbance, and points were fitted via linear regression. The resulting slope yields the β-galactosidase activity.

### Cloning of lrp orthologs

The *lrp *genes (translational start to stop) from *E. coli O157:H7*, *P. mirabilis HI4320 *and *V. cholerae El tor A1552 *were PCR amplified from chromosomal DNA using Pfx DNA polymerase (Invitrogen). The upstream PCR primers (Table S1) contained a consensus *E. coli *ribosome binding site. Fragments were gel purified and cloned into the low-copy pCC1 blunt cloning vector (Epicentre), and transformed into *E. coli EPI300 *per the manufacturer's protocol. As a vector control, an irrelevant ~1360 bp DNA fragment (kanamycin resistance cassette provided by the manufacturer as a ligation control) was inserted into pCC1. Transformants were selected using chloramphenicol and sequence-confirmed. The recombinants pECLRP, pPMLRP and pVCLRP (Table 1) were isolated using Qiagen miniprep columns. The purified plasmids were then electroporated into *E. coli BE3780 *(Table 1) using a BioRad *E. coli *gene pulser and protocol. For experiments with *P. mirabilis *and *V. cholerae *Δ*lrp *strains, which are already chloramphenicol resistant, these plasmids were digested with BsmI to remove the *cat *gene, and we inserted a kanamycin resistance gene PCR amplified from pACYC177.

### Construction of *lacZ *fusions

The promoter regions of the *lrp *and *gltB *geneswere PCR amplified from *E. coli *O157:H7, *Proteus mirabilis *HI4320 and *Vibrio cholerae *El tor type N16969 chromosomal DNA using gene specific primers (Table S1) and Pfx DNA polymerase (Invitrogen). The PCR products were digested with BamHI and SalI and ligated into pBH403, which is a derivative of pKK232-8 and contains a promoterless *lacZ *gene between two bidirectional transcription terminators. The recombinant plasmids (Table 1) were electroporated into *E. coli *BE10.2 and PS2209;*Proteus mirabilis *U6450 and U6450*Δlrp*; and *Vibrio cholerae *El tor strain A1552 and A1552*Δlrp*.

## Authors' contributions

RMB, REL, PKM and ABK contributed to the conception and design of experiments. REL, PKM, BMMV and PS performed the experiments. REL, PKM, ABK and RMB analyzed and interpreted the data, with ABK primarily responsible for statistical analyses. REL and RMB drafted and revised the manuscript, with input from all authors.

## Supplementary Material

Additional file 1 Lrp in gamma proteobacteria (Fig. S1), and table of oligonucleotides used (Table S1). Click here for file

Additional file 2 Genes significantly affected by various Lrp orthologs in *E. coli K*-12. Click here for file

## References

[B1] Liolios K, Tavernarakis N, Hugenholtz P, Kyrpides NC (2006). The Genomes On Line Database (GOLD) v.2: a monitor of genome projects worldwide. Nucleic Acids Res.

[B2] Handelsman J (2004). Metagenomics: application of genomics to uncultured microorganisms. Microbiol Mol Biol Rev.

[B3] Riesenfeld CS, Schloss PD, Handelsman J (2004). Metagenomics: genomic analysis of microbial communities. Annu Rev Genet.

[B4] Rusch DB, Halpern AL, Sutton G, Heidelberg KB, Williamson S, Yooseph S, Wu D, Eisen JA, Hoffman JM, Remington K, Beeson K, Tran B, Smith H, Baden-Tillson H, Stewart C, Thorpe J, Freeman J, Andrews-Pfannkoch C, Venter JE, Li K, Kravitz S, Heidelberg JF, Utterback T, Rogers YH, Falcon LI, Souza V, Bonilla-Rosso G, Eguiarte LE, Karl DM, Sathyendranath S, Platt T, Bermingham E, Gallardo V, Tamayo-Castillo G, Ferrari MR, Strausberg RL, Nealson K, Friedman R, Frazier M, Venter JC (2007). The Sorcerer II Global Ocean Sampling Expedition: Northwest Atlantic through Eastern Tropical Pacific. PLoS Biol.

[B5] Steele HL, Streit WR (2005). Metagenomics: advances in ecology and biotechnology. FEMS Microbiol Lett.

[B6] Field D, Kyrpides N (2007). The positive role of the ecological community in the genomic revolution. Microb Ecol.

[B7] Gill SR, Pop M, Deboy RT, Eckburg PB, Turnbaugh PJ, Samuel BS, Gordon JI, Relman DA, Fraser-Liggett CM, Nelson KE (2006). Metagenomic analysis of the human distal gut microbiome. Science.

[B8] Kennedy J, Marchesi JR, Dobson AD (2007). Metagenomic approaches to exploit the biotechnological potential of the microbial consortia of marine sponges. Appl Microbiol Biotechnol.

[B9] Edwards RA, Rodriguez-Brito B, Wegley L, Haynes M, Breitbart M, Peterson DM, Saar MO, Alexander S, Alexander EC, Rohwer F (2006). Using pyrosequencing to shed light on deep mine microbial ecology. BMC Genomics.

[B10] Duncan MJ (2003). Genomics of oral bacteria. Crit Rev Oral Biol Med.

[B11] Mao F, Su Z, Olman V, Dam P, Liu Z, Xu Y (2006). Mapping of orthologous genes in the context of biological pathways: An application of integer programming. Proc Natl Acad Sci U S A.

[B12] Powell BC, Hutchison CA (2006). Similarity-based gene detection: using COGs to find evolutionarily-conserved ORFs. BMC Bioinformatics.

[B13] Edwards JS, Ibarra RU, Palsson BO (2001). In silico predictions of Escherichia coli metabolic capabilities are consistent with experimental data. Nat Biotechnol.

[B14] Jothi R, Przytycka TM, Aravind L (2007). Discovering functional linkages and uncharacterized cellular pathways using phylogenetic profile comparisons: a comprehensive assessment. BMC Bioinformatics.

[B15] Galbraith SJ, Tran LM, Liao JC (2006). Transcriptome network component analysis with limited microarray data. Bioinformatics.

[B16] Kim H, Hu W, Kluger Y (2006). Unraveling condition specific gene transcriptional regulatory networks in Saccharomyces cerevisiae. BMC Bioinformatics.

[B17] Pournara I, Wernisch L (2007). Factor analysis for gene regulatory networks and transcription factor activity profiles. BMC Bioinformatics.

[B18] Qian J, Lin J, Luscombe NM, Yu H, Gerstein M (2003). Prediction of regulatory networks: genome-wide identification of transcription factor targets from gene expression data. Bioinformatics.

[B19] Wang Y, Joshi T, Zhang XS, Xu D, Chen L (2006). Inferring gene regulatory networks from multiple microarray datasets. Bioinformatics.

[B20] VanBogelen RA, Greis KD, Blumenthal RM, Tani TH, Matthews RG (1999). Mapping regulatory networks in microbial cells. Trends Microbiol.

[B21] Balaji S, Aravind L (2007). The two faces of short-range evolutionary dynamics of regulatory modes in bacterial transcriptional regulatory networks. Bioessays.

[B22] Espinosa V, Gonzalez AD, Vasconcelos AT, Huerta AM, Collado-Vides J (2005). Comparative studies of transcriptional regulation mechanisms in a group of eight gamma-proteobacterial genomes. J Mol Biol.

[B23] Ravcheev DA, Gerasimova AV, Mironov AA, Gelfand MS (2007). Comparative genomic analysis of regulation of anaerobic respiration in ten genomes from three families of gamma-proteobacteria (Enterobacteriaceae, Pasteurellaceae, Vibrionaceae). BMC Genomics.

[B24] Madan Babu M, Teichmann SA, Aravind L (2006). Evolutionary dynamics of prokaryotic transcriptional regulatory networks. J Mol Biol.

[B25] Devos D, Valencia A (2000). Practical limits of function prediction. Proteins.

[B26] Tian W, Skolnick J (2003). How well is enzyme function conserved as a function of pairwise sequence identity?. J Mol Biol.

[B27] Whisstock JC, Lesk AM (2003). Prediction of protein function from protein sequence and structure. Q Rev Biophys.

[B28] Alexander PA, He Y, Chen Y, Orban J, Bryan PN (2007). The design and characterization of two proteins with 88% sequence identity but different structure and function. Proc Natl Acad Sci U S A.

[B29] Price MN, Dehal PS, Arkin AP (2007). Orthologous Transcription Factors in Bacteria Have Different Functions and Regulate Different Genes. PLoS Comput Biol.

[B30] Bulyk ML, McGuire AM, Masuda N, Church GM (2004). A motif co-occurrence approach for genome-wide prediction of transcription-factor-binding sites in Escherichia coli. Genome Res.

[B31] Gelfand MS, Novichkov PS, Novichkova ES, Mironov AA (2000). Comparative analysis of regulatory patterns in bacterial genomes. Brief Bioinform.

[B32] Sandve GK, Drablos F (2006). A survey of motif discovery methods in an integrated framework. Biol Direct.

[B33] Sarai A, Kono H (2005). Protein-DNA recognition patterns and predictions. Annu Rev Biophys Biomol Struct.

[B34] Tan K, McCue LA, Stormo GD (2005). Making connections between novel transcription factors and their DNA motifs. Genome Res.

[B35] Gelfand MS (2006). Evolution of transcriptional regulatory networks in microbial genomes. Curr Opin Struct Biol.

[B36] Robison K, McGuire AM, Church GM (1998). A comprehensive library of DNA-binding site matrices for 55 proteins applied to the complete Escherichia coli K-12 genome. J Mol Biol.

[B37] Thieffry D, Salgado H, Huerta AM, Collado-Vides J (1998). Prediction of transcriptional regulatory sites in the complete genome sequence of Escherichia coli K-12. Bioinformatics.

[B38] Reddy TE, DeLisi C, Shakhnovich BE (2007). Binding site graphs: a new graph theoretical framework for prediction of transcription factor binding sites. PLoS Comput Biol.

[B39] Schones DE, Smith AD, Zhang MQ (2007). Statistical significance of cis-regulatory modules. BMC Bioinformatics.

[B40] Kalir S, Alon U (2004). Using a quantitative blueprint to reprogram the dynamics of the flagella gene network. Cell.

[B41] Liu J, Stormo GD (2005). Combining SELEX with quantitative assays to rapidly obtain accurate models of protein-DNA interactions. Nucleic Acids Res.

[B42] Liu J, Stormo GD (2005). Quantitative analysis of EGR proteins binding to DNA: assessing additivity in both the binding site and the protein. BMC Bioinformatics.

[B43] Liu Z, Mao F, Guo JT, Yan B, Wang P, Qu Y, Xu Y (2005). Quantitative evaluation of protein-DNA interactions using an optimized knowledge-based potential. Nucleic Acids Res.

[B44] Morozov AV, Havranek JJ, Baker D, Siggia ED (2005). Protein-DNA binding specificity predictions with structural models. Nucleic Acids Res.

[B45] Lozada-Chavez I, Janga SC, Collado-Vides J (2006). Bacterial regulatory networks are extremely flexible in evolution. Nucleic Acids Res.

[B46] Tsong AE, Tuch BB, Li H, Johnson AD (2006). Evolution of alternative transcriptional circuits with identical logic. Nature.

[B47] Mayo AE, Setty Y, Shavit S, Zaslaver A, Alon U (2006). Plasticity of the cis-regulatory input function of a gene. PLoS Biol.

[B48] Hidalgo E, Demple B (1997). Spacing of promoter elements regulates the basal expression of the soxS gene and converts SoxR from a transcriptional activator into a repressor. Embo J.

[B49] Kamionka A, Bogdanska-Urbaniak J, Scholz O, Hillen W (2004). Two mutations in the tetracycline repressor change the inducer anhydrotetracycline to a corepressor. Nucleic Acids Res.

[B50] Lin SH, Kovac L, Chin AJ, Chin CC, Lee JC (2002). Ability of E. coli cyclic AMP receptor protein to differentiate cyclic nucelotides: effects of single site mutations. Biochemistry.

[B51] Suiter AM, Banziger O, Dean AM (2003). Fitness consequences of a regulatory polymorphism in a seasonal environment. Proc Natl Acad Sci U S A.

[B52] EcoSal (2007). Escherichia coli and Salmonella cellular and molecular biology.

[B53] Blattner FR, Plunkett G, Bloch CA, Perna NT, Burland V, Riley M, Collado-Vides J, Glasner JD, Rode CK, Mayhew GF, Gregor J, Davis NW, Kirkpatrick HA, Goeden MA, Rose DJ, Mau B, Shao Y (1997). The complete genome sequence of Escherichia coli K-12. Science.

[B54] Sanger_Institute Proteus mirabilis genome sequencing project. http://www.sanger.ac.uk/Projects/P_mirabilis/.

[B55] Heidelberg JF, Eisen JA, Nelson WC, Clayton RA (2000). DNA sequence of both chromosomes of the cholera pathogen Vibrio cholerae. Nature.

[B56] Dick H, Murray RG, Walmsley S (1985). Swarmer cell differentiation of Proteus mirabilis in fluid media. Can J Microbiol.

[B57] Harshey RM (1994). Bees aren't the only ones: swarming in gram-negative bacteria. Mol Microbiol.

[B58] Gardel CL, Mekalanos JJ (1996). Alterations in Vibrio cholerae motility phenotypes correlate with changes in virulence factor expression. Infect Immun.

[B59] Rather PN (2005). Swarmer cell differentiation in Proteus mirabilis. Environ Microbiol.

[B60] Nielsen AT, Dolganov NA, Otto G, Miller MC, Wu CY, Schoolnik GK (2006). RpoS controls the Vibrio cholerae mucosal escape response. PLoS Pathog.

[B61] Matz C, McDougald D, Moreno AM, Yung PY, Yildiz FH, Kjelleberg S (2005). Biofilm formation and phenotypic variation enhance predation-driven persistence of Vibrio cholerae. Proc Natl Acad Sci U S A.

[B62] Martinez-Antonio A, Collado-Vides J (2003). Identifying global regulators in transcriptional regulatory networks in bacteria. Curr Opin Microbiol.

[B63] Brinkman AB, Ettema TJ, de Vos WM, van der Oost J (2003). The Lrp family of transcriptional regulators. Mol Microbiol.

[B64] Calvo JM, Matthews RG (1994). The leucine-responsive regulatory protein, a global regulator of metabolism in Escherichia coli. Microbiol Rev.

[B65] Newman EB, Lin R (1995). Leucine-responsive regulatory protein: a global regulator of gene expression in E. coli. Annu Rev Microbiol.

[B66] Bhagwat SP, Rice MR, Matthews RG, Blumenthal RM (1997). Use of an inducible regulatory protein to identify members of a regulon: application to the regulon controlled by the leucine-responsive regulatory protein (Lrp) in Escherichia coli. J Bacteriol.

[B67] Ernsting BR, Atkinson MR, Ninfa AJ, Matthews RG (1992). Characterization of the regulon controlled by the leucine-responsive regulatory protein in Escherichia coli. J Bacteriol.

[B68] Hung SP, Baldi P, Hatfield GW (2002). Global gene expression profiling in Escherichia coli K12. The effects of leucine-responsive regulatory protein. J Biol Chem.

[B69] Tani TH, Khodursky A, Blumenthal RM, Brown PO, Matthews RG (2002). Adaptation to famine: a family of stationary-phase genes revealed by microarray analysis. Proc Natl Acad Sci U S A.

[B70] Tchetina E, Newman EB (1995). Identification of Lrp-regulated genes by inverse PCR and sequencing: regulation of two mal operons of Escherichia coli by leucine-responsive regulatory protein. J Bacteriol.

[B71] Salgado H, Gama-Castro S, Peralta-Gil M, Diaz-Peredo E, Sanchez-Solano F, Santos-Zavaleta A, Martinez-Flores I, Jimenez-Jacinto V, Bonavides-Martinez C, Segura-Salazar J, Martinez-Antonio A, Collado-Vides J (2006). RegulonDB (version 5.0): Escherichia coli K-12 transcriptional regulatory network, operon organization, and growth conditions. Nucleic Acids Res.

[B72] Salgado H, Santos-Zavaleta A, Gama-Castro S, Peralta-Gil M, Penaloza-Spinola MI, Martinez-Antonio A, Karp PD, Collado-Vides J (2006). The comprehensive updated regulatory network of Escherichia coli K-12. BMC Bioinformatics.

[B73] Chen S, Calvo JM (2002). Leucine-induced dissociation of Escherichia coli Lrp hexadecamers to octamers. J Mol Biol.

[B74] Ernsting BR, Denninger JW, Blumenthal RM, Matthews RG (1993). Regulation of the gltBDF operon of Escherichia coli: how is a leucine-insensitive operon regulated by the leucine-responsive regulatory protein?. J Bacteriol.

[B75] Azam TA, Ishihama A (1999). Twelve species of the nucleoid-associated protein from Escherichia coli. Sequence recognition specificity and DNA binding affinity. J Biol Chem.

[B76] Peterson SN, Dahlquist FW, Reich NO (2007). The role of high affinity non-specific DNA binding by Lrp in transcriptional regulation and DNA organization. J Mol Biol.

[B77] Friedberg D, Plakto JV, Tyler B, Calvo JM (1995). The amino acid sequence of Lrp is highly conserved in four enteric microorganisms. J Bacteriol.

[B78] Leonard PM, Smits SH, Sedelnikova SE, Brinkman AB, de Vos WM, van der Oost J, Rice DW, Rafferty JB (2001). Crystal structure of the Lrp-like transcriptional regulator from the archaeon Pyrococcus furiosus. Embo J.

[B79] Platko JV, Calvo JM (1993). Mutations affecting the ability of Escherichia coli Lrp to bind DNA, activate transcription, or respond to leucine. J Bacteriol.

[B80] de los Rios S, Perona JJ (2007). Structure of the Escherichia coli leucine-responsive regulatory protein Lrp reveals a novel octameric assembly. J Mol Biol.

[B81] Price MN, Dehal PS, Arkin AP (2008). Horizontal gene transfer and the evolution of transcriptional regulation in Escherichia coli. Genome Biol.

[B82] Friedberg D, Midkiff M, Calvo JM (2001). Global versus local regulatory roles for Lrp-related proteins: Haemophilus influenzae as a case study. J Bacteriol.

[B83] Nakamoto T (2006). A unified view of the initiation of protein synthesis. Biochem Biophys Res Commun.

[B84] Hay NA, Tipper DJ, Gygi D, Hughes C (1997). A nonswarming mutant of Proteus mirabilis lacks the Lrp global transcriptional regulator. J Bacteriol.

[B85] Chen CF, Lan J, Korovine M, Shao ZQ, Tao L, Zhang J, Newman EB (1997). Metabolic regulation of lrp gene expression in Escherichia coli K-12. Microbiology.

[B86] Landgraf JR, Wu J, Calvo JM (1996). Effects of nutrition and growth rate on Lrp levels in Escherichia coli. J Bacteriol.

[B87] Willins DA, Ryan CW, Platko JV, Calvo JM (1991). Characterization of Lrp, and Escherichia coli regulatory protein that mediates a global response to leucine. J Biol Chem.

[B88] Tung JS, Knight CA (1972). Relative importance of some factors affecting the electrophoretic migration of proteins in sodium dodecyl sulfate-polyacrylamide gels. Anal Biochem.

[B89] Wang Q, Wu J, Friedberg D, Plakto J, Calvo JM (1994). Regulation of the Escherichia coli lrp gene. J Bacteriol.

[B90] Lloyd G, Landini P, Busby S (2001). Activation and repression of transcription initiation in bacteria. Essays Biochem.

[B91] Rhodius VA, Busby SJ (1998). Positive activation of gene expression. Curr Opin Microbiol.

[B92] Borst DW, Blumenthal RM, Matthews RG (1996). Use of an in vivo titration method to study a global regulator: effect of varying Lrp levels on expression of gltBDF in Escherichia coli. J Bacteriol.

[B93] Paul L, Blumenthal RM, Matthews RG (2001). Activation from a distance: roles of Lrp and integration host factor in transcriptional activation of gltBDF. J Bacteriol.

[B94] Paul L, Mishra PK, Blumenthal RM, Matthews RG (2007). Integration of regulatory signals through involvement of multiple global regulators: control of the Escherichia coli gltBDF operon by Lrp, IHF, Crp, and ArgR. BMC Microbiol.

[B95] Landick R, Oxender DL (1985). The complete nucleotide sequences of the Escherichia coli LIV-BP and LS-BP genes. Implications for the mechanism of high-affinity branched-chain amino acid transport. J Biol Chem.

[B96] Haney SA, Platko JV, Oxender DL, Calvo JM (1992). Lrp, a leucine-responsive protein, regulates branched-chain amino acid transport genes in Escherichia coli. J Bacteriol.

[B97] Thaw P, Sedelnikova SE, Muranova T, Wiese S, Ayora S, Alonso JC, Brinkman AB, Akerboom J, van der Oost J, Rafferty JB (2006). Structural insight into gene transcriptional regulation and effector binding by the Lrp/AsnC family. Nucleic Acids Res.

[B98] Quay SC, Dick TE, Oxender DL (1977). Role of transport systems in amino acid metabolism: leucine toxicity and the branched-chain amino acid transport systems. J Bacteriol.

[B99] Bouvier J, Gordia S, Kampmann G, Lange R, Hengge-Aronis R, Gutierrez C (1998). Interplay between global regulators of Escherichia coli: effect of RpoS, Lrp and H-NS on transcription of the gene osmC. Mol Microbiol.

[B100] Lin R, Ernsting B, Hirshfield IN, Matthews RG, Neidhardt FC, Clark RL, Newman EB (1992). The lrp gene product regulates expression of lysU in Escherichia coli K-12. J Bacteriol.

[B101] Rhee KY, Parekh BS, Hatfield GW (1996). Leucine-responsive regulatory protein-DNA interactions in the leader region of the ilvGMEDA operon of Escherichia coli. J Biol Chem.

[B102] Munch R, Hiller K, Grote A, Scheer M, Klein J, Schobert M, Jahn D (2005). Virtual Footprint and PRODORIC: an integrative framework for regulon prediction in prokaryotes. Bioinformatics.

[B103] Wong ML, Medrano JF (2005). Real-time PCR for mRNA quantitation. Biotechniques.

[B104] Kessler D, Herth W, Knappe J (1992). Ultrastructure and pyruvate formate-lyase radical quenching property of the multienzymic AdhE protein of Escherichia coli. J Biol Chem.

[B105] Kessler D, Leibrecht I, Knappe J (1991). Pyruvate-formate-lyase-deactivase and acetyl-CoA reductase activities of Escherichia coli reside on a polymeric protein particle encoded by adhE. FEBS Lett.

[B106] Knappe J, Sawers G (1990). A radical-chemical route to acetyl-CoA: the anaerobically induced pyruvate formate-lyase system of Escherichia coli. FEMS Microbiol Rev.

[B107] Jung IL, Phyo KH, Kim IG (2005). RpoS-mediated growth-dependent expression of the Escherichia coli tkt genes encoding transketolases isoenzymes. Curr Microbiol.

[B108] Lange R, Hengge-Aronis R (1991). Growth phase-regulated expression of bolA and morphology of stationary-phase Escherichia coli cells are controlled by the novel sigma factor sigma S. J Bacteriol.

[B109] Membrillo-Hernandez J, Lin EC (1999). Regulation of expression of the adhE gene, encoding ethanol oxidoreductase in Escherichia coli: transcription from a downstream promoter and regulation by fnr and RpoS. J Bacteriol.

[B110] Vanoni MA, Curti B (1999). Glutamate synthase: a complex iron-sulfur flavoprotein. Cell Mol Life Sci.

[B111] Vanoni MA, Dossena L, van den Heuvel RH, Curti B (2005). Structure-function studies on the complex iron-sulfur flavoprotein glutamate synthase: the key enzyme of ammonia assimilation. Photosynth Res.

[B112] Wiese DE, Ernsting BR, Blumenthal RM, Matthews RG (1997). A nucleoprotein activation complex between the leucine-responsive regulatory protein and DNA upstream of the gltBDF operon in Escherichia coli. J Mol Biol.

[B113] Hommais F, Krin E, Coppee JY, Lacroix C, Yeramian E, Danchin A, Bertin P (2004). GadE (YhiE): a novel activator involved in the response to acid environment in Escherichia coli. Microbiology.

[B114] Wang Q, Frye JG, McClelland M, Harshey RM (2004). Gene expression patterns during swarming in Salmonella typhimurium: genes specific to surface growth and putative new motility and pathogenicity genes. Mol Microbiol.

[B115] Pul U, Wurm R, Wagner R (2007). The role of LRP and H-NS in transcription regulation: involvement of synergism, allostery and macromolecular crowding. J Mol Biol.

[B116] Chen C, Newman EB (1998). Comparison of the sensitivities of two Escherichia coli genes to in vivo variation of Lrp concentration. J Bacteriol.

[B117] Beach MB, Osuna R (1998). Identification and characterization of the fis operon in enteric bacteria. J Bacteriol.

[B118] Mallik P, Pratt TS, Beach MB, Bradley MD, Undamatla J, Osuna R (2004). Growth phase-dependent regulation and stringent control of fis are conserved processes in enteric bacteria and involve a single promoter (fis P) in Escherichia coli. J Bacteriol.

[B119] Chen J, Hsueh HM, Delongchamp R, Lin CJ, Tsai CA (2007). Reproducibility of microarray data: a further analysis of microarray quality control (MAQC)data. BMC Bioinformatics.

[B120] Shi L, Reid LH, Jones WD, Shippy R, Warrington JA, Baker SC, Collins PJ, de Longueville F, Kawasaki ES, Lee KY, Luo Y, Sun YA, Willey JC, Setterquist RA, Fischer GM, Tong W, Dragan YP, Dix DJ, Frueh FW, Goodsaid FM, Herman D, Jensen RV, Johnson CD, Lobenhofer EK, Puri RK, Schrf U, Thierry-Mieg J, Wang C, Wilson M, Wolber PK, Zhang L, Amur S, Bao W, Barbacioru CC, Lucas AB, Bertholet V, Boysen C, Bromley B, Brown D, Brunner A, Canales R, Cao XM, Cebula TA, Chen JJ, Cheng J, Chu TM, Chudin E, Corson J, Corton JC, Croner LJ, Davies C, Davison TS, Delenstarr G, Deng X, Dorris D, Eklund AC, Fan XH, Fang H, Fulmer-Smentek S, Fuscoe JC, Gallagher K, Ge W, Guo L, Guo X, Hager J, Haje PK, Han J, Han T, Harbottle HC, Harris SC, Hatchwell E, Hauser CA, Hester S, Hong H, Hurban P, Jackson SA, Ji H, Knight CR, Kuo WP, LeClerc JE, Levy S, Li QZ, Liu C, Liu Y, Lombardi MJ, Ma Y, Magnuson SR, Maqsodi B, McDaniel T, Mei N, Myklebost O, Ning B, Novoradovskaya N, Orr MS, Osborn TW, Papallo A, Patterson TA, Perkins RG, Peters EH, Peterson R, Philips KL, Pine PS, Pusztai L, Qian F, Ren H, Rosen M, Rosenzweig BA, Samaha RR, Schena M, Schroth GP, Shchegrova S, Smith DD, Staedtler F, Su Z, Sun H, Szallasi Z, Tezak Z, Thierry-Mieg D, Thompson KL, Tikhonova I, Turpaz Y, Vallanat B, Van C, Walker SJ, Wang SJ, Wang Y, Wolfinger R, Wong A, Wu J, Xiao C, Xie Q, Xu J, Yang W, Zhang L, Zhong S, Zong Y, Slikker W (2006). The MicroArray Quality Control (MAQC) project shows inter- and intraplatform reproducibility of gene expression measurements. Nat Biotechnol.

[B121] Grainger DC, Hurd D, Goldberg MD, Busby SJ (2006). Association of nucleoid proteins with coding and non-coding segments of the Escherichia coli genome. Nucleic Acids Res.

[B122] Lucchini S, Rowley G, Goldberg MD, Hurd D, Harrison M, Hinton JC (2006). H-NS mediates the silencing of laterally acquired genes in bacteria. PLoS Pathog.

[B123] Navarre WW, Porwollik S, Wang Y, McClelland M, Rosen H, Libby SJ, Fang FC (2006). Selective silencing of foreign DNA with low GC content by the H-NS protein in Salmonella. Science.

[B124] Oshima T, Ishikawa S, Kurokawa K, Aiba H, Ogasawara N (2006). Escherichia coli histone-like protein H-NS preferentially binds to horizontally acquired DNA in association with RNA polymerase. DNA Res.

[B125] Arfin SM, Long AD, Ito ET, Tolleri L, Riehle MM, Paegle ES, Hatfield GW (2000). Global gene expression profiling in Escherichia coli K12. The effects of integration host factor. J Biol Chem.

[B126] Mangan MW, Lucchini S, Danino V, Croinin TO, Hinton JC, Dorman CJ (2006). The integration host factor (IHF) integrates stationary-phase and virulence gene expression in Salmonella enterica serovar Typhimurium. Mol Microbiol.

[B127] Bradley MD, Beach MB, de Koning AP, Pratt TS, Osuna R (2007). Effects of Fis on Escherichia coli gene expression during different growth stages. Microbiology.

[B128] Kelly A, Goldberg MD, Carroll RK, Danino V, Hinton JC, Dorman CJ (2004). A global role for Fis in the transcriptional control of metabolism and type III secretion in Salmonella enterica serovar Typhimurium. Microbiology.

[B129] Marr C, Geertz M, Huett MT, Muskhelishvili G (2008). Dissecting the logical types of network control in gene expression profiles. BMC Syst Biol.

[B130] Wade JT, Reppas NB, Church GM, Struhl K (2005). Genomic analysis of LexA binding reveals the permissive nature of the Escherichia coli genome and identifies unconventional target sites. Genes Dev.

[B131] Grainger DC, Overton TW, Reppas N, Wade JT, Tamai E, Hobman JL, Constantinidou C, Struhl K, Church G, Busby SJ (2004). Genomic studies with Escherichia coli MelR protein: applications of chromatin immunoprecipitation and microarrays. J Bacteriol.

[B132] Neidhardt FC, Bloch PL, Smith DF (1974). Culture medium for enterobacteria. J Bacteriol.

[B133] Serra-Moreno R, Acosta S, Hernalsteens JP, Jofre J, Muniesa M (2006). Use of the lambda Red recombinase system to produce recombinant prophages carrying antibiotic resistance genes. BMC Mol Biol.

[B134] Lin-Chao S, Cohen SN (1991). The rate of processing and degradation of antisense RNAI regulates the replication of ColE1-type plasmids in vivo. Cell.

[B135] Khodursky AB, Bernstein JA, Peter BJ, Rhodius V, Wendisch VF, Zimmer DP (2003). Escherichia coli spotted double-strand DNA microarrays: RNA extraction, labeling, hybridization, quality control, and data management. Methods Mol Biol.

[B136] Berger JA, Hautaniemi S, Jarvinen AK, Edgren H, Mitra SK, Astola J (2004). Optimized LOWESS normalization parameter selection for DNA microarray data. BMC Bioinformatics.

[B137] Cui X, Kerr MK, Churchill GA (2003). Transformations for cDNA microarray data. Stat Appl Genet Mol Biol.

[B138] Tusher VG, Tibshirani R, Chu G (2001). Significance analysis of microarrays applied to the ionizing radiation response. Proc Natl Acad Sci U S A.

[B139] Platko JV, Willins DA, Calvo JM (1990). The ilvIH operon of Escherichia coli is positively regulated. J Bacteriol.

[B140] NCBI BLink list of Lrp orthologs/paralogs. http://www.ncbi.nlm.nih.gov/sutils/blink.cgi?pid=16128856.

[B141] Jeong KS, Xie Y, Hiasa H, Khodursky AB (2006). Analysis of pleiotropic transcriptional profiles: a case study of DNA gyrase inhibition. PLoS Genet.

[B142] Kerr MK, Martin M, Churchill GA (2000). Analysis of variance for gene expression microarray data. J Comput Biol.

