# Supplementary material for: Limited functional conservation of a global regulator among related bacterial genera: Lrp in Escherichia, Proteus and Vibrio
Source: BMC Microbiol. 2008 Apr 11;8:60. doi: 10.1186/1471-2180-8-60 (PMC2374795; doi:10.1186/1471-2180-8-60)
Supplement: Additional file 1 — Lrp in gamma proteobacteria (Fig. S1), and table of oligonucleotides used (Table S1). [file 1471-2180-8-60-S1.pdf]

Supplementary material for BMC Microbiology, 2008:

Limited Functional Conservation of a Global Regulator Among Related Bacterial Genera: Lrp in *Escherichia*, *Proteus* and *Vibrio*

Robert E Lintner, Pankaj K Mishra, Poonam Srivastava, Betsy M Martinez-Vaz, Arkady B Khodursky and Robert M Blumenthal

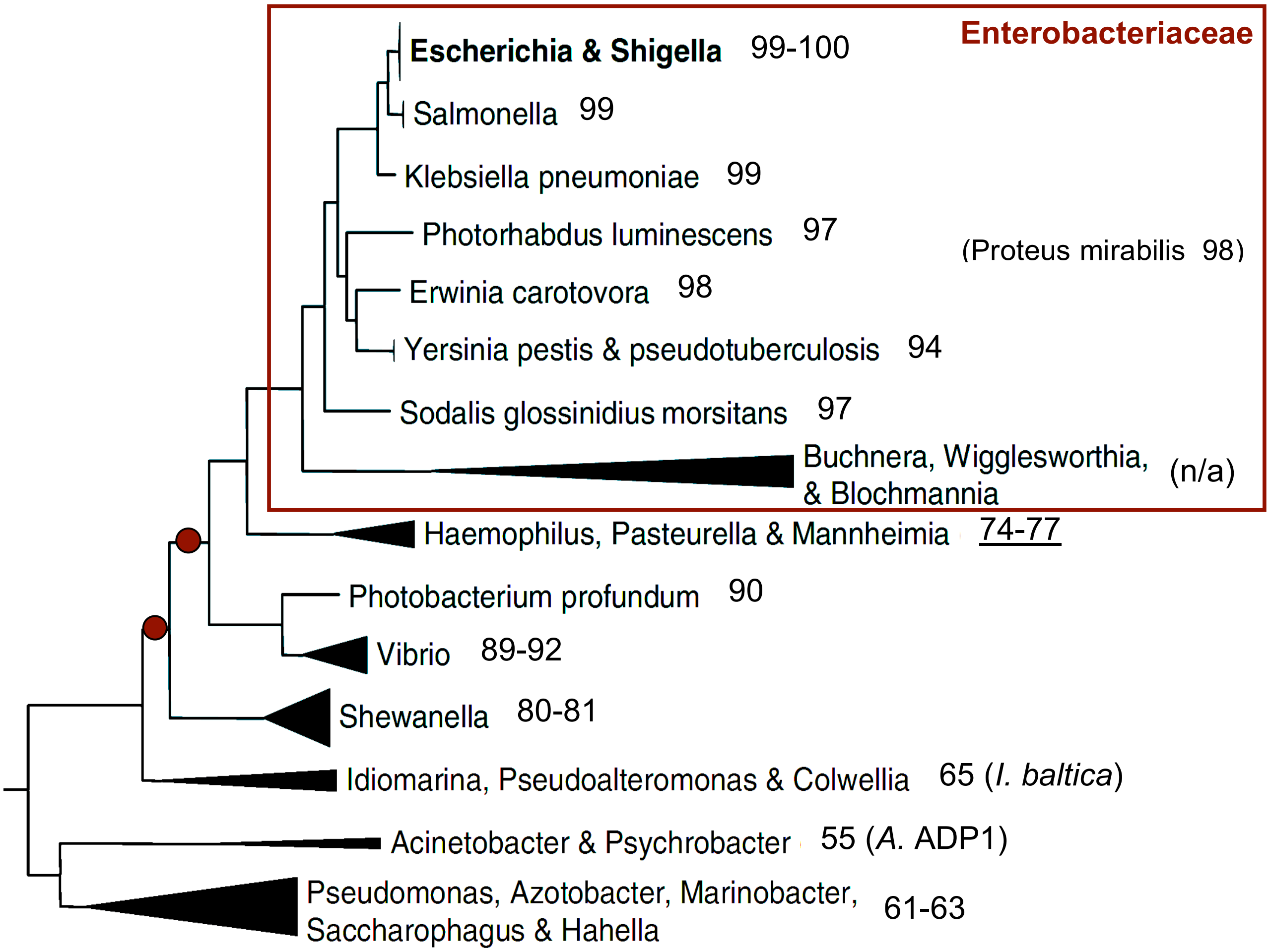

Figure S1. Comparison of Lrp conservation to that of the host bacterial genome. The tree is adapted from Figure 2 of [1], which was generated from concatenated alignments of 74 highly-conserved proteins. The numbers indicate percent identity (at the level of amino acid sequence) to *E. coli K*-12 Lrp, as indicated by the BLink feature of NCBI [2]. The Pasteurellaceae (Haemophilus, Pasteurella and Mannheimia) appear to be an outgroup with respect to Lrp, having lower Lrp similarity to *E. coli* Lrp than would be predicted by the overall similarity of the 74 highly-conserved proteins. The Vibrionaceae, more distant overall from *E. coli* than the Pasteurellaceae, nevertheless have Lrp proteins substantially more closely related to the *E. coli* ortholog. In *E. coli*, *Vibrio cholerae* and *Proteus mirabilis* (not shown here; another of the Enterobacteriaceae with *E. coli*), Lrp is a global regulator (see text and references therein). In contrast, Lrp controls a small number of genes in *Haemophilus influenzae* [3]. The filled circles indicate possible positions for the ancestral strain in which Lrp served as a global regulator. As it is not yet known if Lrp serves as a global regulator in Shewanella or Photobacterium, it is also possible that Vibrionaceae are the outgroup.

Supplementary Table 1. **Oligonucleotides used**

**Primers – *lrp* genes**	(all shown 5' → 3'; consensus rbs underlined)
eclrp1	GAAGGAGATATACATATGGTAGATAGCAAGAAGCG
eclrp2	TTAGCGCGTCTTAATAACCAGACGATTACTC
pmlrp1	GAAGGAGATATACATATGATTGATAATAAAAAACGTC
pmlrp2	TTAGCGTGTTTTAATCACTAGGCG
vclrp1	GAAGGAGATATACATATGGTGGATAGTTATAAGAAGC
vclrp2	TTAGCGAGTTTTAATCACTAATTGGTTGG
**Primers – *gltB* promoters**	
O157gltb1	CGGGATCCCATAATCACATAAATCACTTTTGCTTATC
O157gltb2	ACGCGTCGACAGCGGATTTCCAACTTATCG
pmgltb1	CGGGATCCGGGTATTTATAAAAACAATTTATAGAGGTA
pmgltb2	ACGCGTCGACTTGCTATCTCGGTTTTTTATTTTGC
vc2376-1	CGGGATCCAGCACTCCCTGCTGGC
vc2376-2	ACGCGTCGACTGATGGGGATTCATCCACTTCC
vc2373-1	CGGGATCCATTGGTCACAAATTTTAATCCCTCG
vc2373-2	ACGCGTCGACCAGTTATTCCCGCCACG
**Primers – *lrp* promoters**	
ecolrp+69-1	CGGGATCCTGACGATTTTGTTAACAATTTGTGCAA
ecolrp+69-2	ACGCGTCGACACTAGCACGGTAAACATGGTATTTAC
pmlrp+69-1	CGGGATCCTAGCGATAATATTTTAACTATTTTAGCAATA
pmlrp+69-2	ACGCGTCGACAAAAGCACTATCCTATGCCC
Vc lrpfull-1	CGGGATCCTCT TCA GTT TGT GGA AAA TCT G
Vc lrpfull-2	ACGCGTCGACCTTCCTTGCAAAAAAATATACTAC

**Primers – QRT-PCR**	
erart1	ATGGCTATCGACGAAAAC
erart2	GGTTTTACCGGAAGATTCC
prart1	ATGGCTATTGATGAAAACAAAC
prart2	GAAGATTCAGGGCCATAG
vrart1	ATGGACGAGAATAAACAGAAG
vrart2	GTGTCGTTTTACCCGAAG
elrprt1	ATGGTAGATAGCAAGAAGC
elrprt2	GATGCATCCAGATAATGGG
plrprt1	ATGATTGATAATAAAAAACGTCCG
plrprt2	GGGTTTAATAGCGCAGTATA
vlrprt1	ATGGTGGATAGTTATAAGAAGC
vlrprt2	CTGAGGGTTCAATAATGCA
eadh1	ATGGCTGTTACTAATGTCGCT
eadh2	GGTTTTTGATCACTTTATCTTCG
padh1	AACGTTACTGAACTCAATGATCT
padh2	ATTGTAGATATATTCTGAAGCAAAG
vadh1	ATGCCTGTTACTAATCTGGCT
vadh2	ATAAACTCAGAAGCAAAGTGGT
eglt1	ATGACACGCAAACCCCG
eglt2	CGCACTACCTTGTGGCTAGG
pglt1	TGCTCTATCAAAAAACAGCGG
pglt2	CTGAAAAAAGCGCGTTGG
vglt1	ACTCAGTGCGGTTGCGTC
vglt2	TCACAACCCTGACCGCC

Literature cited in Supplement:

1. Price MN, Dehal PS, Arkin AP: **Horizontal gene transfer and the evolution of transcriptional regulation in Escherichia coli**. *Genome Biol* 2008, **9**(1):R4.
2. Wheeler DL, Barrett T, Benson DA, Bryant SH, Canese K, Chetvernin V, Church DM, Dicuccio M, Edgar R, Federhen S *et al*: **Database resources of the National Center for Biotechnology Information**. *Nucleic Acids Res* 2008, **36**(Database issue):D13-21.
3. Friedberg D, Midkiff M, Calvo JM: **Global versus local regulatory roles for Lrp-related proteins: Haemophilus influenzae as a case study**. *J Bacteriol* 2001, **183**(13):4004-4011.
